# Innovative MXene/SilMA-Based Conductive Bioink for Three Dimensional Bioprinting of Neural Stem Cell Spheroids in Neural Tissue Engineering

**DOI:** 10.1021/acsami.4c19373

**Published:** 2025-02-07

**Authors:** Yu-Chun Yeh, Pin-Yuan Chen, Ko-Ting Chen, I-Chi Lee

**Affiliations:** 1 Department of Biomedical Engineering and Environmental Sciences, National Tsing Hua University, Hsinchu 300044, Taiwan; 2 Department of Neurosurgery, Chang Gung Memorial Hospital, Keelung Branch, Keelung 20401, Taiwan; 3 Department of Neurosurgery, Chang Gung Memorial Hospital at Linkou, Taoyuan 333, Taiwan

**Keywords:** neural stem cells (NSCs), SilMA, conductive bioink, MXene-SP, 3D bioprinting, electrical stimulation

## Abstract

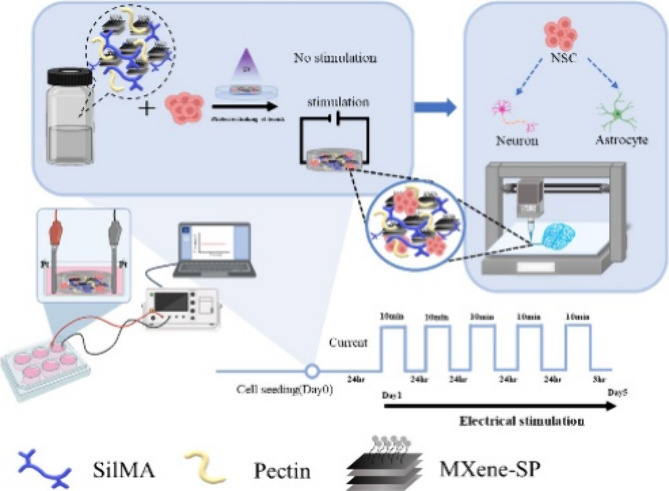

Conductive bioinks, integrated with 3D bioprinting and electrical stimulation, are essential for advancing neural tissue engineering. This study developed a SilMA/Pectin/MXene-soybean phospholipids (SP) bioink, where SilMA (silk fibroin modified with glycidyl methacrylate) provides a structural base, pectin enhances printability and shear-thinning properties, and MXene-SP improves conductivity through superior dispersibility. Increasing pectin and MXene-SP concentrations reduced the hydrogel’s Young’s modulus, promoting neural stem cell (NSC) differentiation into neurons. Electrochemical analyses revealed that higher MXene-SP levels decreased impedance and increased redox current, while conductivity measurements showed improved performance compared to unmodified MXene. NSCs encapsulated in the bioink achieved maximum proliferation under electrical stimulation at 300 μA for 10 min daily over 5 days. Neuronal differentiation positively correlated with MXene-SP concentration and stimulation intensity. Synaptic activity and vesicle recycling, assessed using FM1–43 dye, were significantly enhanced under electrical stimulation. This study successfully developed a biocompatible conductive bioink capable of inducing neuronal differentiation. Electrical stimulation further promoted cell proliferation, neuronal differentiation, and enhanced synaptic function. This bioink shows great potential for future applications in neural tissue engineering.

## 1 Introduction

Spinal cord injury (SCI) is a serious neurological condition that causes neuron necrosis and axonal damage, disrupting cellular connections and leading to sensory and motor function loss below the injury site. This loss significantly reduces patients’ quality of life and imposes a substantial economic burden on society. SCI occurs in two stages: primary and secondary injury. Primary injury involves direct or indirect mechanical damage, leading to spinal compression, neuron and oligodendrocyte damage, vascular disruption, edema, inflammation, and cell necrosis. Secondary injury induces further damage through inflammatory responses, attracting macrophages, microglia, and astrocytes to the injury site. This results in glial cell proliferation, cytokine release, and scar formation, exacerbating pathology and hindering neuronal regeneration.^1^ Current treatments for SCI, including surgical decompression and stabilization, anti-inflammatory medications, and rehabilitation, are primarily focused on minimizing secondary injury and facilitating recovery.^2^ 3D bioprinting bioinks offer flexibility and ease of shaping, creating a porous structure with mechanical properties similar to natural tissue. These bioinks can be injected or implanted at the SCI site, acting as scaffolds that support neural cell growth, promote axonal regeneration, and inhibit scar formation.^3^

Silk fibroin (SF) is a natural biomaterial known for its excellent mechanical properties, elasticity, biocompatibility, and biodegradability. After modification with glycidyl methacrylate (GMA), it forms SilMA, a bioink suitable for 3D bioprinting. Our previous studies have demonstrated that the addition of Pectin, an anionic water-soluble polysaccharide derived from natural sources, enhances the printability of SilMA.^4^ In recent years, conductive hydrogels have been shown to facilitate physiological functions through electrical stimulation, with significant applications in the central nervous system (CNS), angiogenesis, cardiac function, and skeletal muscle contraction.^5^ The regenerative capacity of the CNS is limited, as glial scars that form at injury sites hinder axonal regeneration and disrupt electrical signal transmission.^6^ Conductive hydrogels are regarded as highly promising for neural tissue regeneration. Combined with electrical stimulation, they can further promote neuronal differentiation, increase neurite extension in response to electric fields, and enable endogenous and exogenous cells to exchange electrical signals, thereby enhancing synaptic functionality.

Carbon-based conductive hydrogels, which feature delocalized π bonds enabling electron flow, offer excellent conductivity. Common materials include graphene oxide, carbon nanotubes, and MXene. Graphene, known for its robust mechanical properties, large surface area, electrical conductivity, and biocompatibility, has become a popular additive in conductive hydrogels. Graphene-based composite hydrogels have significant applications in regenerative medicine, particularly for neural and cardiac tissue repair.^7^ Alexandre Xavier Mendes and colleagues studied the effects of electrical currents on PC12 cell proliferation using GelMA/GO composite hydrogels. They found that adding graphene oxide (GO) increased capacitance density, reduced impedance, and, along with electrical stimulation, significantly boosted PC12 cell metabolic activity and DNA content compared to GelMA alone.^8^ Yangnan Hu et al. developed a nerve conduit incorporating rGO, BDNF, and GelMA with a Morpho butterfly wing-like topography for peripheral nerve repair. The results showed that the wing-like topography effectively induced neural stem cells (NSCs) to differentiate into neurons. Furthermore, the nerve conduit containing reduced graphene oxide (rGO) further promoted the generation of differentiated neurons. Immunofluorescence staining confirmed that the rGO/BDNF/GelMA combination significantly increased the proportion of NSCs differentiating into neurons and enhanced neurite outgrowth. After implanting the conduit into a 10 mm defect in the rat sciatic nerve for 8 weeks of repair, successful axonal regeneration and myelin sheath formation were observed.^9^ A significant limitation of carbon nanomaterials in biomedical applications is their poor water solubility, which results in material aggregation and reduced conductivity. In recent years, surface modification has been widely used to improve the dispersion of carbon-based materials in aqueous environments. MXene, a family of materials with the general formula M_n+1_X_n_T_x_ (where n = 1–4), has gained attention for its hydrophilic properties and excellent conductivity, making it promising for biomedical applications.^10^ Derived from the MAX phase (M_n+1_AX_n_), MXenes are synthesized by selectively etching away the A layer from the MAX phase, leaving behind M-X layers with strong covalent bonds and functionalized with groups like −OH, -O, or -F to improve hydrophilicity. MXenes are recognized for their electrical conductivity, stability, and biocompatibility, making them suitable for various biomedical uses.^11^ Compared to other two-dimensional materials such as graphene and black phosphorus, MXene offers superior hydrophilicity, which has led to its widespread application in the biomedical field. Its conductive properties are also gaining increasing attention in neural tissue engineering. In 2022, Yige Li et al. studied Ti_3_C_2_Tx MXene’s impact on neural circuit maturation. NSCs cultured on MXene films showed similar biocompatibility and significantly longer axons (41.75 ± 2.75 μm) compared to those on TCPS (29.84 ± 2.19 μm), indicating MXene promotes axonal growth. Although synapse numbers did not differ, the frequency of spontaneous excitatory postsynaptic currents increased, suggesting MXene enhances synaptic transmission.^12^ Hui Liao et al. developed a freeze-resistant, flexible, and self-healing MXene nanocomposite hydrogel (MNOH) by polymerizing MXene with polyacrylamide (PAAm) and poly(vinyl alcohol) (PVA). This hydrogel showed excellent freeze resistance, moisture retention, self-repair, and stable electrical conductivity, making it suitable for flexible wearable strain sensors, such as electronic skin and healthcare monitoring.^13^ In 2022, Jiaying Cai et al. investigated a microgroove-structured GelMA-MXene conductive hydrogel nerve conduit designed to promote the directional growth of neurons for SCI repair. Animal experiments confirmed that the GelMA-MXene conductive hydrogel facilitated neuronal differentiation, demonstrating its feasibility for use in SCI repair.^14^ In 2024, Hao Wei et al. developed a 3D conductive MXene-Matrigel hydrogel to enhance NSC proliferation and differentiation. By cross-linking MXene with Matrigel, a matrix rich in laminin and collagen, the hydrogel provided a conductive environment that significantly promoted NSC growth and neuronal differentiation, highlighting its potential for neural tissue.^15^

3D bioprinting enables precise control over the volume, structure, and shape of hydrogels, simulating *in vivo* cell growth conditions such as migration, differentiation, and proliferation. This technique is particularly valuable in tissue engineering, where bioinks must meet criteria such as mechanical properties, degradability, biocompatibility, and printability. Incorporating conductive materials can enhance the gelation, biological compatibility, and conductivity of the hydrogels, further expanding their potential in tissue engineering applications.^16^ Chen et al., developed a 3D-printable conductive biomimetic scaffold (Gel/HA/PL1) incorporating PEDOT into GelMA/HAMA to promote NSC differentiation and assist in SCI repair. treatment with the Gel/HA/PL1 hydrogel combined with NSCs resulted in improved motor function, as evidenced by elevated scores in the Basso, Beattie, and Bresnahan (BBB) locomotor test. Histological analysis revealed higher expression of Tuj-1 and MAP2, alongside reduced glial scar formation, compared to the Gel/HA group.^17^ In addition, Liu et al., investigated chitosan, hyaluronic acid derivatives, and matrigel bioink, using 3D printing to replicate the neural tissue scaffold of spinal cord white matter. The study showed that the bioprinted scaffold created a favorable microenvironment for NSC growth and differentiation. Directional printing facilitated neuronal and oligodendrocyte differentiation along the longitudinal axis. *In vivo* experiments showed that bioprinted scaffolds enhanced axonal regeneration, reduced glial scarring, and improved locomotor recovery in SCI model rats, offering a versatile strategy for neural tissue engineering in regenerative medicine.^18^

**Scheme 1:**
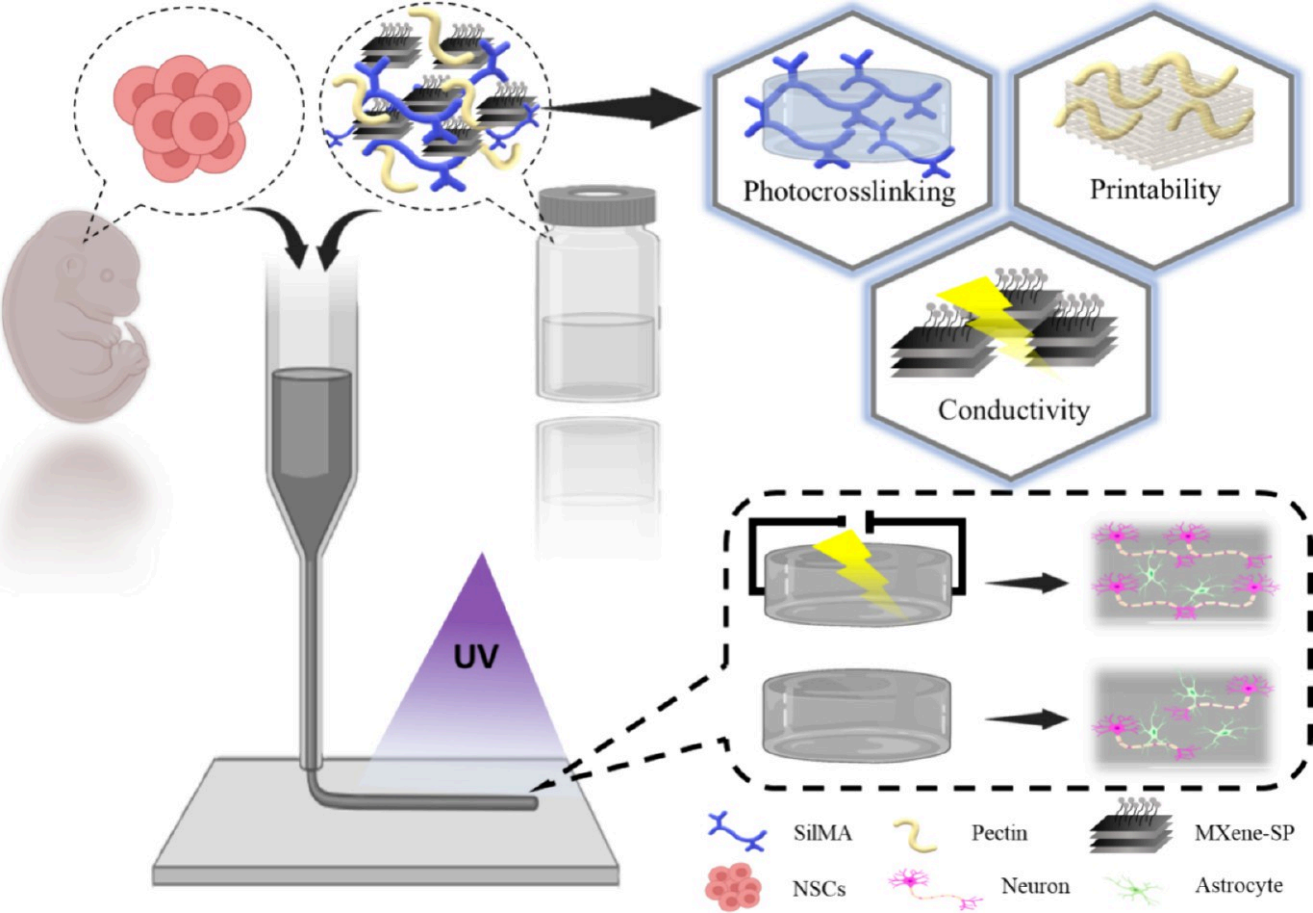
Illustration of Conductive Bioink Composition, Properties, and NSC Spheroid-Laden 3D Bioprinting with Electrical Stimulation

In summary, the development of 3D-printable conductive bioinks combined with appropriate electrical stimulation parameters will significantly contribute to the advancement of neural tissue engineering. This study prepared a series of conductive bioinks based on SilMA/Pectin/MXene-SP to investigate their physical properties and printability. Modifying MXene with soybean phospholipids (SP) is expected to improve the biocompatibility and dispersion of bioinks. NSCs were encapsulated to simulate the microenvironment of the spinal cord or brain. The differentiation of NSCs within the various hydrogels was compared and analyzed. Subsequently, different levels of electrical stimulation were applied to mimic biological electrical signals, allowing for the examination of the differentiation ratios of neurons and astrocytes, as well as synaptic functionality in these environments. Scheme 1 shows the illustration of conductive bioink composition, properties, and NSC spheroid-laden 3D bioprinting with electrical stimulation. In the future, it is anticipated that the SilMA/Pectin/MXene-SP conductive bioinks will serve as an alternative for spinal cord injury repair and have applications in neural tissue engineering.

## 2 Materials and Methods

### 2.1 Preparation of SF and SilMA

The isolation of SF and synthesis of SilMA followed our previous protocol.^4^ Silk cocoons were chopped, boiled in 0.05 M sodium carbonate solution at 100 °C for 30 min to remove sericin, then washed, stirred, and dried overnight. A 9.3 M bromide solution was added to the dried fibers, heated at 60 °C for 4 h. Glycidyl Methacrylate (GMA) at 0.4 M was added (0.7 g GMA per gram of SF). The resulting SilMA solution was dialyzed for 5 days, centrifuged twice at 9,000 rpm, and freeze-dried for 72 h.

Pectin, derived from citrus peels (Sigma-Aldrich, U.S.A), has a molecular weight range of 30,000–100,000 g/mol and an esterification degree of 70–75% (dry basis).^19,20^

### 2.2 Nuclear Magnetic Resonance (NMR) Spectroscopy and Fourier Transform Infrared (FTIR) Analysis

A 1% (w/v) solution of SF and SilMA was prepared in 1 mL of D_2_O and analyzed using a 600 MHz NMR spectrometer (AVANCE 500 NMR, BRUKER, Germany). Proton (1H) NMR spectra were recorded, and each sample was analyzed in triplicate to assess modifications and determine the degree of substitution. Additionally, ATR-FTIR spectroscopy was performed to identify functional groups by analyzing 10 mg of each material in the range of 400–4000 cm^–1^.

### 2.3 Preparation and Characterization of MXene and MXene-SP

To prepare MXene, mix 37% HCl with deionized water and stir for 5 min. Add LiF, stir until dissolved, and cover with plastic wrap. Add 1 g of MAX, cover with two layers of plastic wrap, and heat in an oil bath at 500 rpm for 24 h. Transfer the mixture to 50 mL centrifuge tubes, rinse with 2 M HCl at 4 °C, and centrifuge three times at 8000 rpm for 5 min each. Rinse with deionized water at 4 °C, centrifuge until neutral pH, sonicate for 30 min, and centrifuge at 3500 rpm for 20 min to obtain the MXene supernatant. Freeze at −20 °C for 1 day and freeze-dry for 48 h. For MXene-SP, sonicate MXene in anhydrous ethanol for 30 min. Dissolve soybean phospholipids in chloroform, add to the MXene solution, and sonicate for 5 min. Remove the solvent using a rotary evaporator at 60 °C for 10 min, then vacuum for 30 min. Dissolve MXene-SP in PBS, sonicate for 10 min, transfer to centrifuge tubes, and store at 4 °C.

To characterize the morphology and charge of MXene, 20 ppm solutions of MXene and MXene-SP were sonicated for 30 min. 10 μL of each solution was deposited on a carbon-coated copper grid and dried at 50 °C for 24 h before being observed using a transmission electron microscope (TEM). Zeta potential measurements were also taken on 20 ppm solutions to assess their interfacial charge using a Zetasizer Nano ZS (Malvern, UK).

### 2.4 Gelation Test of Different Concentrations of SilMA/Pectin/MXene-SP Hydrogels

Twenty wt% SilMA solutions were prepared with 2% or 3% Pectin and 0.5% or 1% MXene. Each mixture was supplemented with 0.5% photoinitiator (PI2959, Sigma-Aldrich, U.S.A). A 200 μL portion of each solution was placed into 5 mm diameter, 4 mm height molds and subjected to UV LED photopolymerization (BK-LineCure100 mm, 365 nm) for 30 or 60 s. The gelation and morphology of the hydrogels were then assessed.

### 2.5 Scanning Electron Microscopy (SEM) Images

The surface morphology of the hydrogels was analyzed using scanning electron microscopy (SEM). The hydrogels were prepared according to the described protocol and subsequently frozen in liquid nitrogen. After freezing, the samples were fractured and affixed to a metal substrate using SEM-conductive adhesive tape. The samples were then sputter-coated with platinum for 60 s and examined using an SEM instrument (JSM-7610F, JEOL, Japan).

### 2.6 Porosity Test

Porosity was determined using the solvent replacement method based on our previous study.^4^ Dried hydrogels (Wd) were weighed, immersed in absolute ethanol (purity >99.9%) for 24 h, then blotted and reweighed (Ws). The dimensions of the hydrogels were also measured. Porosity was calculated using the following formula
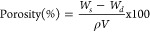
1Here, ρ represents the density of absolute ethanol at 25 °C (0.79 g/cm^3^), and V is the volume of the hydrogel after swelling. Wd and Ws denote the weights of the dried and swollen hydrogels, respectively.

### 2.7 Swelling Ratio

The swelling behavior of hydrogels depends on polymer chain length, cross-linking density, and mesh size. We follow our previous method, preparing series of hydrogels with varying compositions. W_1_ is the weight of freshly synthesized hydrogels, and W_2_ is the weight after 24 h of immersion in 1 mL of distilled water at 37 °C. The swelling ratio is calculated using this formula.:
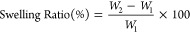
2

### 2.8 Rheological Properties Evaluation and Compressive Strength Testing

Hydrogel solutions of SilMA/pectin/MXene-SP with varying concentrations are prepared and fabricated as previously described. Compression strength is evaluated using a stress testing machine (Rapid TA, China) at a strain rate of 20% per minute. The compression modulus is determined from the slope of the linear portion of the strain curve between 0 and 5%.

Rheological properties of the hydrogels were assessed using a DH rheometer (TA Instruments) with a 60 mm 0.5281° cone plate (Peltier plate aluminum). For shear-thinning tests, the material solution was prepared and tested over a shear rate range of 1–100 1/s. During frequency sweep analysis, 5 mL of the solution was prepared and exposed to UV light for 30 s to form hydrogels. The frequency was varied from 0.01 to 10 Hz with 0.5% strain. If the storage modulus and loss modulus (*G*′, *G*′′) were within the linear range, a strain sweep was conducted with a frequency of 1 Hz and strain ranging from 0.01% to 100%.

### 2.9 Printability Test

The stability of a hydrogel extruded via extrusion-based 3D printing is essential for preserving the printed structure’s integrity. To evaluate bioink printability, tests were conducted to assess material extrusion continuity, pattern design, and the Pr value. A modified method from the literature was used, where the grid perimeter was set as Pr = 1, and the average score was calculated to determine the printability and consistency of the printed patterns.^21^

Bioinks with varying compositions of SilMA/Pectin/MXene-SP solutions were prepared and heated to 37 °C. A CAD file for the design was created using SolidWorks. One milliliter of the bioink was loaded into a custom syringe with a needle tip. Extrusion parameters were configured in Ultimaker Cura to match the nozzle size (22g, 0.413 mm), and the corresponding G-code file was generated. Printing was performed with a pressure range of 50–100 mm/min and a printing speed of 1–30 mm/s. A 2 × 2 grid pattern was printed and subsequently cured under UV light (800 mW/cm^2^) for varying times, with a stacking thickness ranging from 0 to 500 μm. The Pr value, calculated as the following formula, compared the printed grid areas using ImageJ. Pr values less than, equal to, or greater than 1 indicated insufficient, optimal, or excessive gelation, respectively, with printability improving as Pr approaches 1.
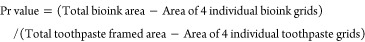
3

### 2.10 Electrochemical Characteristics and Conductivity of Series of SilMA/Pectin/MXene-SP Hydrogel

To measure the electrical impedance of a series of bioinks, 150 μL of each bioink was injected into a 96-well plate with copper foil tape (20 × 3 mm) attached to each side. After curing the bioinks with UV for 30 s, 100 μL of PBS was added to the hydrogel. Electrical impedance measurements were performed using a dual-electrode system over a frequency range of 0.01 Hz to 100,000 Hz with an applied AC voltage of 0.01 V. The resulting data were analyzed by fitting Nyquist plots and Bode diagrams.

Cyclic voltammetry (CV) is a widely used analytical technique in electrochemical systems. It involves varying the electrode potential to determine the oxidation or reduction potential of a sample. By performing a linear scan of the potential in both positive and negative directions, the oxidation potential is obtained from the negative-to-positive scan, and the reduction potential is obtained from the positive-to-negative scan. The resulting current versus potential plot reveals peaks and current values that indicate the electrochemical properties of the analyte. In this study, experimental parameters were adapted from Dong Nyoung Heo et al.,^22^ using a method similar to electrochemical impedance analysis. CV was performed with a scan rate of 50 mV/s over a potential range of −0.6 to 0.6 V, with 20 repeated scans to obtain the CV curves.

To measure the conductivity of the hydrogel, 250 μL of the bioinks was injected into a mold and cured with light for 30 s. Platinum electrodes (3 × 1 × 0.1 cm) were then placed on either side of the hydrogel. Impedance measurements were conducted at a fixed frequency of 100,000 Hz with an applied AC voltage of 0.01 V. The resistance obtained was used to calculate the conductivity using the following formula, where σ represents the hydrogel conductivity, h is the distance between the electrodes, R is the resistance, and D is the diameter of the hydrogel cylinder.
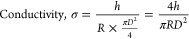
4

### 2.11 NSC Isolation

Primary cortical NSCs were isolated from embryonic day 16 (ED16) Wistar rat cortices after CO_2_ euthanasia, following a modified protocol from our previous study.^23^ The procedures were approved by the IACUC of National Tsing Hua University (No. 109060–2). Pregnant rats were sterilized, and their embryonic cortices were collected, centrifuged, and purified by removing the supernatant and blood vessels. The tissue was dissociated and cultured in a T25 flask with 20 ng/mL bFGF, incubated at 37 °C and 5% CO_2_. Neurospheres formed within 3–4 days, after which the medium was changed for further purification and subculturing.

### 2.12 Biocompatibility Test of Series of Hydrogels

Series of bioinks solutions containing 1.0 × 10^5^ L929 fibroblasts were solidified by light exposure and incubated in 5% CO_2_. After 24 h, biocompatibility was assessed using Live/Dead and MTT assays. The Live/Dead assay employed Calcein AM and Ethidium homodimer-1 to evaluate cell viability. Calcein AM fluoresces green in live cells, while Ethidium homodimer-1 fluoresces red in dead or damaged cells. Samples were incubated with PBS and culture medium containing 1:2000 Calcein AM and 1:500 Ethidium homodimer-1 (ThermoFisher Scientific) for 30 min, followed by confocal microscopy (LSM 800, ZEISS). Fluorescence at 488 and 561 nm excitation was used to detect green (515 nm) and red (635 nm) signals. Cell viability was quantified using ImageJ software.

For the MTT assay, after 24 h, MTT solution was added for 3 h and analyzed at O.D. 570 using an ELISA reader. NSC proliferation was evaluated using CCK-8 at 24 and 72 h, with absorbance measured at O.D. 450.

NSC viability in various hydrogel compositions was also evaluated. Around 500 NSC spheroids were mixed with 100 μL bioinks and solidified using light exposure. Live/Dead and CCK-8 assays were performed after different culture periods. For the CCK-8 assay, following incubation with the hydrogel at specific time points, 50 μL of CCK-8 solution was added to the culture medium and incubated for 3 h. The absorbance was measured at O.D. 450 using an ELISA reader.

### 2.13 Immunostaining for the Differentiation of NSCs in Hydrogels with/without Electrical Stimulation

NSC differentiation in hydrogels was evaluated by immunostaining after 5 days of culture for the groups without electrical stimulation. In addition, for the groups with electrical stimulation, immunostaining was performed after 3 days of culture, followed by 5 consecutive days of electrical stimulation.

The culture medium was removed and cells were washed three times with PBS. Cells were fixed and permeabilized with 70% methanol for 15 min, then washed with PBS. Primary antibodies (MAP2 at 1:1000 and GFAP at 1:1000, both from Sigma-Aldrich) diluted in 10% BSA and Maleic acid (1:100) were applied overnight at 4 °C. Following washes with PBS, secondary antibodies (AP181R at 1:100, AP187F at 1:250) and Hoechst (1:2000) were added for 2 h at room temperature. After washing, cells were mounted and imaged using a confocal microscope. Images were analyzed with ImageJ software, with 3–5 neurospheres counted per condition, and mean and standard deviation were calculated using SigmaPlot software.

### 2.14 Statistical Analysis

The experimental data and images are presented as the mean ± standard deviation or standard error of the mean for each independent experiment. Statistical analyses were performed using Student’s *t* test and analysis of variance (ANOVA), with data analyzed using SigmaPlot software. Statistical significance is indicated by asterisks: *, *p* < 0.05; **, *p* < 0.01; ***, *p* < 0.001.

## 3 Results

### 3.1 Characterization of SF and SilMA by ^1^H NMR and FTIR

**Figure 1:**
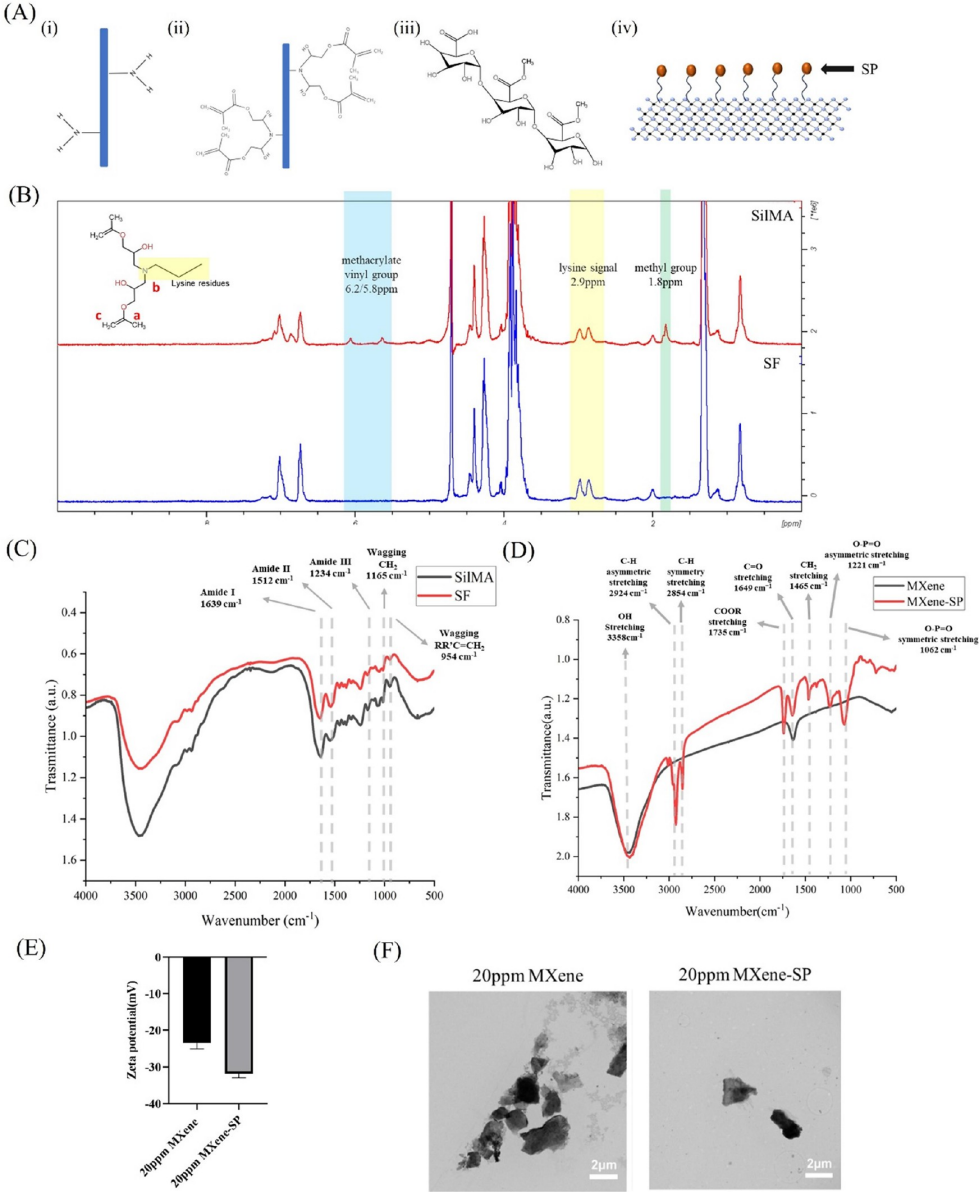
(A) Chemical structures and schematic representations of the materials. (i) SF. (ii) SilMA. (iii) Pectin. (iv) illustration of MXene-SP (B) ^1^H NMR spectrum of SF and SilMA (C) FTIR spectrum of SF and SilMA. (D) FTIR spectrum of MXene and MXene-SP. (E) Zeta potential of MXene and MXene-SP. (F) TEM images of MXene and MXene-SP at 20 ppm.

Figure 1(A) shows the chemical structures and schematic representations of the materials used, including SF, SilMA, Pectin, and MXene-SP. SilMA is synthesized through a substitution reaction where the primary amine (−NH_2_) on the lysine residues of SF reacts with glycidyl methacrylate (GMA) via ring-opening. By comparing the chemical shifts of SF before and after modification, the presence of methacrylate groups in the modified SilMA is observed at δ = 6–6.2 ppm and δ = 5.6–5.8 ppm, corresponding to position c in the SilMA structure shown in Figure 1(B). Additionally, a peak at δ = 1.8 ppm indicates the methyl signal from GMA, as seen at position a in Figure 1(B). The signals between 3.5–4 ppm correspond to lysine and other proteins in SF, as shown at position b in Figure 1(B). These results confirm the successful grafting of GMA onto SF. Based on previous literature, the degree of substitution for SilMA was calculated to be 35.4%.^24^

FTIR spectroscopy is commonly used to analyze molecular structures, also could be used to observe changes in protein composition. SF primarily consists of proteins made up of amino acids linked by amide bonds. From the FTIR spectra of SF and SilMA shown in Figure 1(C), characteristic peaks for SilMA can be observed at wavenumbers 954 and 1168 cm^–1^, which are higher than those in SF. These peaks correspond to the wagging stretching vibrations of CH_2_ and RR’C = CH_2_ from the methacrylate vinyl group in GMA. Additionally, peaks at 1234 cm^–1^, 1512 cm^–1^, and 1639 cm^–1^ represent the Amide III C–N stretching, Amide II N–H bending, and Amide I C = O stretching, respectively. These findings confirm that GMA has been successfully grafted onto SF to form SilMA.

### 3.2 Characterization of MXene and MXene-SP by FTIR, Zeta Potential, and TEM

To enhance bioink biocompatibility and dispersion, we modified MXene with SP and conducted property analyses to assess these improvements. Figure 1(D) presents the FTIR spectra of MXene and MXene-SP. Based on previous literature, the FTIR spectrum of SP shows characteristic peaks at 1062 and 1221 cm^–1^, corresponding to the symmetric and antisymmetric stretching of PO_2_ (phosphorus dioxide) in the lecithin structure. Additionally, a peak at 1465 cm^–1^ represents CH_2_ stretching. The peak at 1649 cm^–1^ is attributed to C = O stretching, which is present in both MXene and SP. The peak at 1735 cm^–1^ corresponds to COOR (ester carbonyl) stretching. Finally, peaks at 1645 cm^–1^, 2854 cm^–1^, and 2924 cm^–1^ represent CH_2_ scissoring vibrations, symmetric CH_2_ stretching, and asymmetric CH_2_ stretching, respectively. These characteristic peaks indicate that MXene-SP exhibits the functional groups of SP, confirming the successful modification of MXene with SP.^25,26^

Figure 1(E) shows the TEM images of MXene and MXene-SP. The TEM images reveal significant agglomeration in MXene, whereas MXene-SP exhibits more uniform dispersion. Further quantification using ImageJ software indicates that the average size of MXene is 2.61 ± 0.34 μm, while MXene-SP has a smaller size of 2.35 ± 0.15 μm. This difference in size suggests that the larger size observed in MXene may be attributed to its tendency to aggregate more readily compared to MXene-SP.

In order to enhance the stability of MXene in water, MXene was modified with SP in this study, which provides steric hindrance through its organic chains, thereby improving MXene’s stability in physiological environments. Previous studies have revealed that particles with a zeta potential between 0 and ±5 mV tend to aggregate easily; those with a potential between ±10 and ±30 mV exhibit slight stability; and particles with a zeta potential between ±30 and ±40 mV demonstrate moderate stability.^27^ As shown in Figure 1(E), MXene has a zeta potential of −23.4 ± 1.37 mV, which increases to −31.8 ± 0.94 mV after modification with SP. This increase in surface potential is attributed to the negatively charged phosphate groups on the soybean phospholipid, which, when attached to MXene, result in a more negative potential and are hypothesized to enhance the stability of MXene-SP in aqueous solutions. Figure 1(F) presents the TEM images of MXene and MXene-SP. The images show that MXene displays noticeable aggregation, whereas MXene-SP is more uniformly dispersed. Quantification using ImageJ revealed that the size of MXene is 2.61 ± 0.34 μm, while MXene-SP measures 2.35 ± 0.15 μm.

### 3.3 Gelation of Series of SilMA-Based Hydrogels

**Figure 2:**
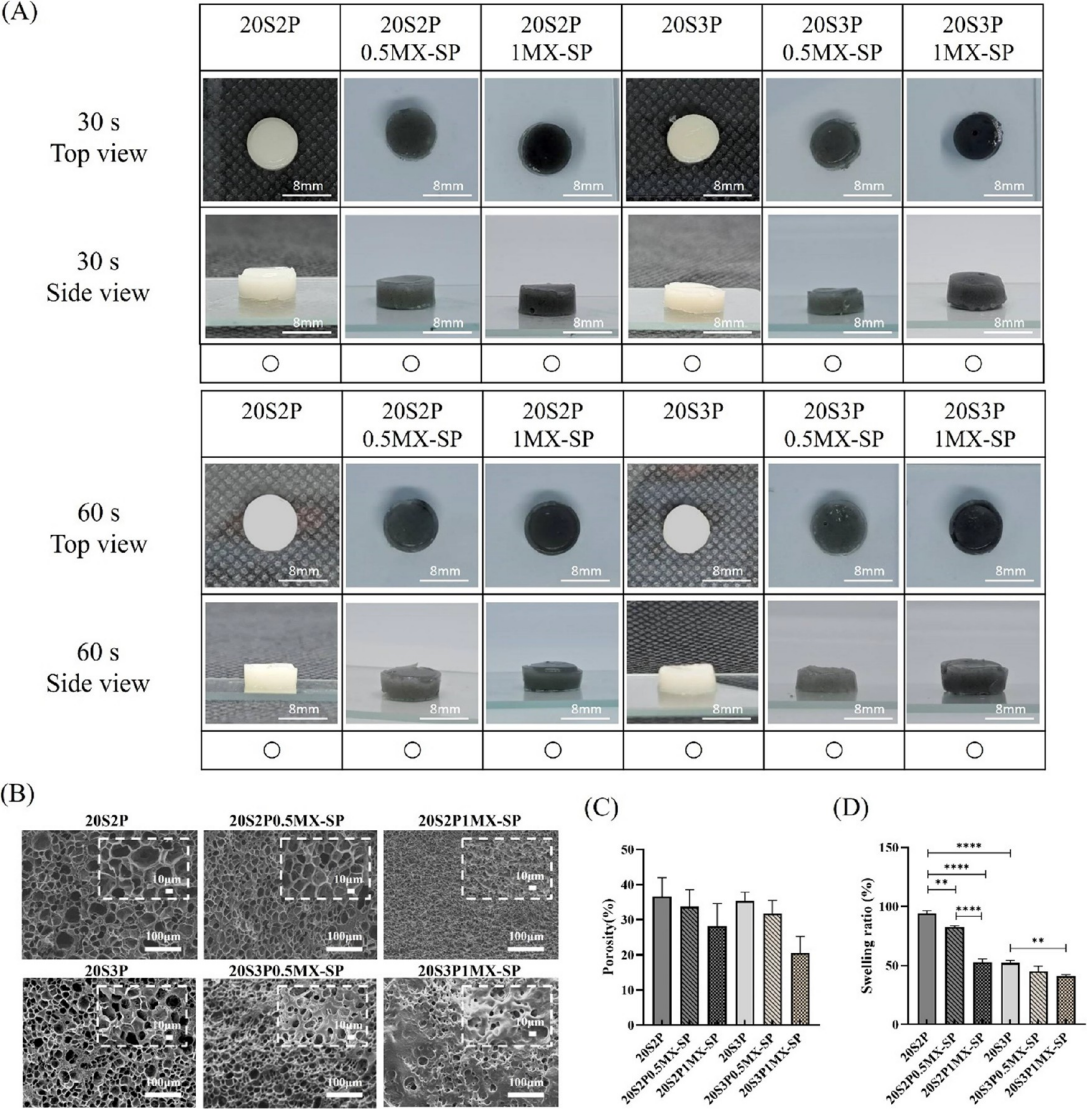
(A) Gelation of series of SilMA/Pectin/MXene-SP hydrogel at different UV curing time. (B) SEM image of series of SilMA/Pectin/MXene-SP hydrogel. (C) Porosity of series of SilMA/Pectin/MXene-SP hydrogel. (D) Swelling ratio of series of SilMA/Pectin/MXene-SP hydrogel.

The photoinitiator (PI, Irgacure 2959, Sigma-Aldrich, USA) was dissolved in a series of bioinks. Upon exposure to UV light at a wavelength of 365 nm, the PI absorbs energy, generating free radicals. These radicals then react with the unsaturated C = C double bonds in SilMA, initiating a radical chain reaction that proceeds through multiple linkages with other SilMA chains before eventually terminating. Bioink samples were injected into PDMS cylindrical molds (10 mm × 3 mm) and exposed to UV light at 365 nm with an intensity of 80 mW/cm^2^ for 30, 60, or 90 s, respectively, to observe gelation after photocuring. According to previous studies,^4^ increasing the Pectin concentration enhances the viscosity of the bioink but reduces the structural stability of the SilMA hydrogel after photocuring. In this study, it is considered that the inclusion of MXene increases opacity, which subsequently affects the cross-linking efficiency. Bioink group abbreviations will be used consistently throughout the text; please refer to Table 1 for a full list of abbreviations. To identify the appropriate SilMA/Pectin hydrogel composition and MXene incorporation, we initially tested combinations of 10% and 15% SilMA with 1% Pectin as the base, varying the MXene concentration and UV exposure time to investigate the impact of MXene on gelation behavior. Figure S1(A) shows that the gelation process is influenced by the MXene concentration, and at a concentration of 3 mg/mL, gelation was not successful even with 90 s of UV exposure. Figure S1(B) demonstrates that increasing the SilMA concentration slightly improves gelation. Additionally, the rheological data in Figure S1(C) indicate that as the MXene concentration increases, the hydrogel’s *G*′ decreases with increasing shear strain. For the overall mechanical properties, we ultimately increased the concentrations of Pectin and SilMA and selected 1 mg/mL of MXene as the highest concentration for the system. Then, Figure S2 shows the gelation states of bioinks 15S2P, 15S2P0.5MX, and 15S2P1MX after 30 s of photocuring, where the internal structure appears soft and collapsed. Increasing the SilMA concentration to 20% improved structural stability. However, with Pectin increased to 3% and MXene to 1 mg/mL, the 20S3P1MX hydrogel showed a collapsed structure even after 30 and 60 s of photocuring, indicating incomplete curing. We hypothesize that this results from MXene’s uneven distribution and opacity, which may interfere with SilMA radical chain linkages. To test if structural stability could be improved, Figure 2(A) compares the gelation of SP-modified MXene hydrogel groups. The results show that, compared to the unmodified 20S3P1MX group, the 20S3P1MX-SP bioink maintained a stable cylindrical shape after 30 s of photocuring. Based on these findings, we selected the 20% SilMA series with SP-modified MXene, as shown in Figure 2(A), for further analysis and application.

**Table 1:** Abbreviations for Series of Bioink Compositions

Bioink composition	Abbreviations
15% SilMA + 2% Pectin	15S2P
15% SilMA + 2% Pectin +0.5 mg/mL MXene	15S2P0.5MX
15% SilMA + 2% Pectin +1 mg/mL MXene	15S2P1MX
20% SilMA + 1% Pectin	20S1P
20% SilMA + 2% Pectin	20S2P
20% SilMA + 2% Pectin +0.5 mg/mL MXene	20S2P0.5MX-SP
20% SilMA + 2% Pectin +1 mg/mL MXene	20S2P1MX-SP
20% SilMA + 2% Pectin +0.5 mg/mL MXene-SP	20S2P0.5MX-SP
20% SilMA + 2% Pectin +1 mg/mL MXene-SP	20S2P1MX-SP
20% SilMA + 3% Pectin	20S3P
20% SilMA + 3% Pectin +0.5 mg/mL MXene-SP	20S3P0.5MX-SP
20% SilMA + 3% Pectin +1 mg/mL MXene-SP	20S3P1MX-SP

### 3.4 SEM Images, Porosity, and Swelling Ratio of Series of Hydrogels

To examine the internal structure of the SilMA/Pectin/MXene-SP hydrogel series, SEM was used to analyze as shown in Figure 2(B). When comparing pure SilMA hydrogel with Pectin addition groups of 20S1P, 20S2P, and 20S3P hydrogels, all displayed a porous structure. The SEM images clearly showed that as Pectin concentration increased, the pore size decreased, and the number of pores increased. This suggests that adding pectin enhances intermolecular interactions within the hydrogel, making the structure more compact. Next, we observed the groups with addition of MXene-SP, which showed slightly smaller pores than those without MXene-SP. This may be due to the MXene-SP nanosheets occupying some of the original hydrogel pores.

The porosity of the hydrogel series was further assessed by immersing the samples in absolute ethanol for 24 h. Absolute ethanol was chosen due to its low surface tension and high permeability, allowing it to uniformly penetrate the hydrogel’s fine pores without altering the hydrogel’s structure or properties. Figure 2(C) presents the porosity percentages for the SilMA/Pectin/MXene-SP hydrogel series. Results indicated that the 20S hydrogel alone exhibited a porosity of 56.33 ± 2.08%. As the Pectin concentration increased, a trend of decreasing porosity was observed. Additionally, at the same Pectin concentration, porosity decreased with increasing MXene-SP concentration. The 20S2P0.5MX-SP group showed a porosity of 33.75 ± 3.94%, which further dropped to 28.10 ± 5.31% as the MXene-SP concentration reached 1 mg/mL. Similarly, in the 20S3P0.5MX-SP and 20S3P1MX-SP groups, porosity decreased from 31.74 ± 3.06% to 20.55 ± 3.85%. This reduction in porosity is likely due to the MXene-SP nanosheets occupying portions of the hydrogel pores, as well as increased hydrogen bonding interactions between materials, which create a denser network with increasing MXene-SP and Pectin concentrations. These results align with observations from the SEM images.

To further simulate the swelling behavior of hydrogels in a physiological environment, we immersed the hydrogels in D-PBS for 24 h. Figure 2(C) shows the swelling ratio of series of hydrogels. The results indicate a decreasing trend in the swelling ratio as Pectin and MXene-SP concentrations increase. This outcome aligns with the SEM observations and porosity results, suggesting that when the porosity of the hydrogel decreases, water molecules may encounter increased surface tension within the smaller pores, which limits effective diffusion and thereby reduces the swelling capacity of the hydrogel.

### 3.5 Mechanical Properties of Series of Hydrogels

**Figure 3:**
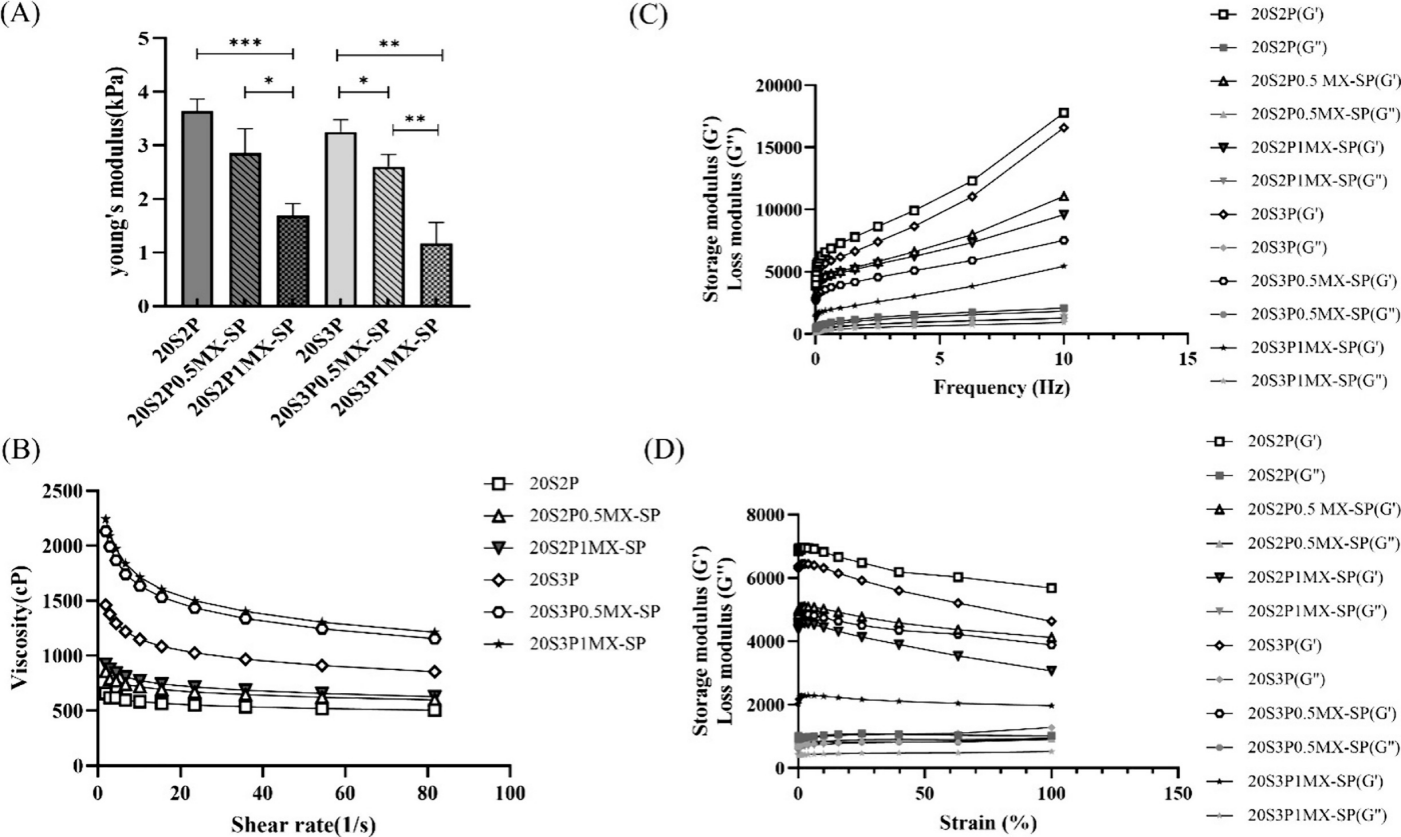
Mechanical characterization of series of SilMA/Pectin/MXene-SP bioinks. (A) Compressive modulus. (B) Viscosity measurement of the hydrogels at a constant shear rate of 200 1/s. (C) Storage modulus and loss modulus of hydrogels at different frequencies. (D) Strain sweeps of hydrogels at frequency of 10 Hz.

Figure 3(A) shows the Young’s modulus of hydrogels with varying concentrations of Pectin and MXene-SP, while maintaining a fixed SilMA concentration of 20%. The results demonstrate that increasing the Pectin concentration from 2% to 3% reduces the Young’s modulus from 3.64 ± 0.18 kPa to 3.25 ± 0.18 kPa, indicating that Pectin contributes to the softening of the hydrogel matrix. Additionally, an increase in MXene-SP concentration also results in a significant decrease in Young’s modulus: 20S2P0.5MX-SP and 20S2P1MX-SP exhibit Young’s moduli of 2.86 ± 0.37 kPa and 1.69 ± 0.18 kPa, respectively, while 20S3P0.5MX-SP and 20S3P1MX-SP decrease from 2.6 ± 0.18 kPa to 1.17 ± 0.32 kPa. These findings align with the results of the gelation experiments, suggesting that the light absorption by MXene-SP affects the photopolymerization of the hydrogel, thereby impacting its Young’s modulus.

According to previous studies by Nic D. Leipzig and colleagues on the influence of substrate stiffness on NSC differentiation, NSCs more readily differentiate into neurons in softer substrates (E < 1 kPa) but tend toward astrocytic differentiation in stiffer substrates (E = 1–10 kPa).^28^ Given that the modulus range of natural spinal cord tissue is 0.1–3 kPa, the conductive hydrogels developed in this study, 20S2P1MX-SP and 20S3P1MX-SP, with Young’s moduli of 1.69 ± 0.18 kPa and 1.17 ± 0.31 kPa, respectively, are expected to simulate the modulus of natural tissue and potentially promote neuronal differentiation.

### 3.6 Shear-Thinning Testing and Rheological Analysis of Series of Bioinks

Figure 3(B) illustrates the shear-thinning behavior of a series of bioinks, which reflects the viscosity changes of the material during extrusion in subsequent 3D printing. The results show that as the Pectin concentration increases, the 20S3P hydrogel exhibits more pronounced shear-thinning properties compared to 20S2P and 20S1P. Additionally, a higher concentration of MXene-SP also enhances the bioink viscosity. This increase in viscosity is likely due to hydrogen bonding between the hydroxyl groups on MXene-SP and the SilMA/Pectin matrix. We also reasonably hypothesize that increased concentrations of Pectin and MXene-SP will contribute to maintaining the shape fidelity during 3D printing.

The storage modulus (*G*′) and loss modulus (*G*′′) are essential rheological parameters that describe a material’s elasticity and viscosity. *G*′ measures the energy stored under stress, reflecting elasticity, while *G*′′ represents energy dissipated, indicating viscosity. When *G*′ > *G*′′, the material behaves more like a solid; when *G*′′ > *G*′, it behaves more like a liquid, suggesting structural instability. Rheological tests, including frequency and strain sweeps, examine how *G*′ and *G*′′ vary with frequency and strain, respectively, to assess the material’s viscoelastic properties.^29^ As shown in Figure 3(C), both *G*′ and *G*′′ increase with frequency across all bioink groups, indicating that as angular frequency rises, the capacities for both energy storage and dissipation increase, with *G*′ consistently remaining greater than *G*′′. The lower *G*′ value observed for 20S1P compared to 20S2P and 20S3P is likely due to limited intermolecular interactions between 1% Pectin and SilMA. As the Pectin concentration increases, additional intermolecular forces enhance the photo-cross-linking network of SilMA, thereby increasing the hydrogel’s storage modulus. However, at 20S3P, an excess of Pectin seems to interfere with SilMA’s photo-cross-linking, causing a reduction in *G*′. Additionally, the presence of MXene-SP also decreases *G*′, likely due to its light-absorbing effect partially obstructing SilMA’s photo-cross-linking, thus impacting the storage modulus. In addition, Figure 3(D) presents the strain sweep results. With increasing strain, all groups exhibit a slight decrease in *G*′ and a stable or slightly increasing *G*′′, though no significant changes are observed within this operational range. Overall, both frequency and strain sweeps confirm that *G*′ exceeds *G*′′ in this cross-linked hydrogel series, indicating that these hydrogels maintain solid-like characteristics.

### 3.7 Printability of the Series of SilMA-Based Bioinks

**Figure 4:**
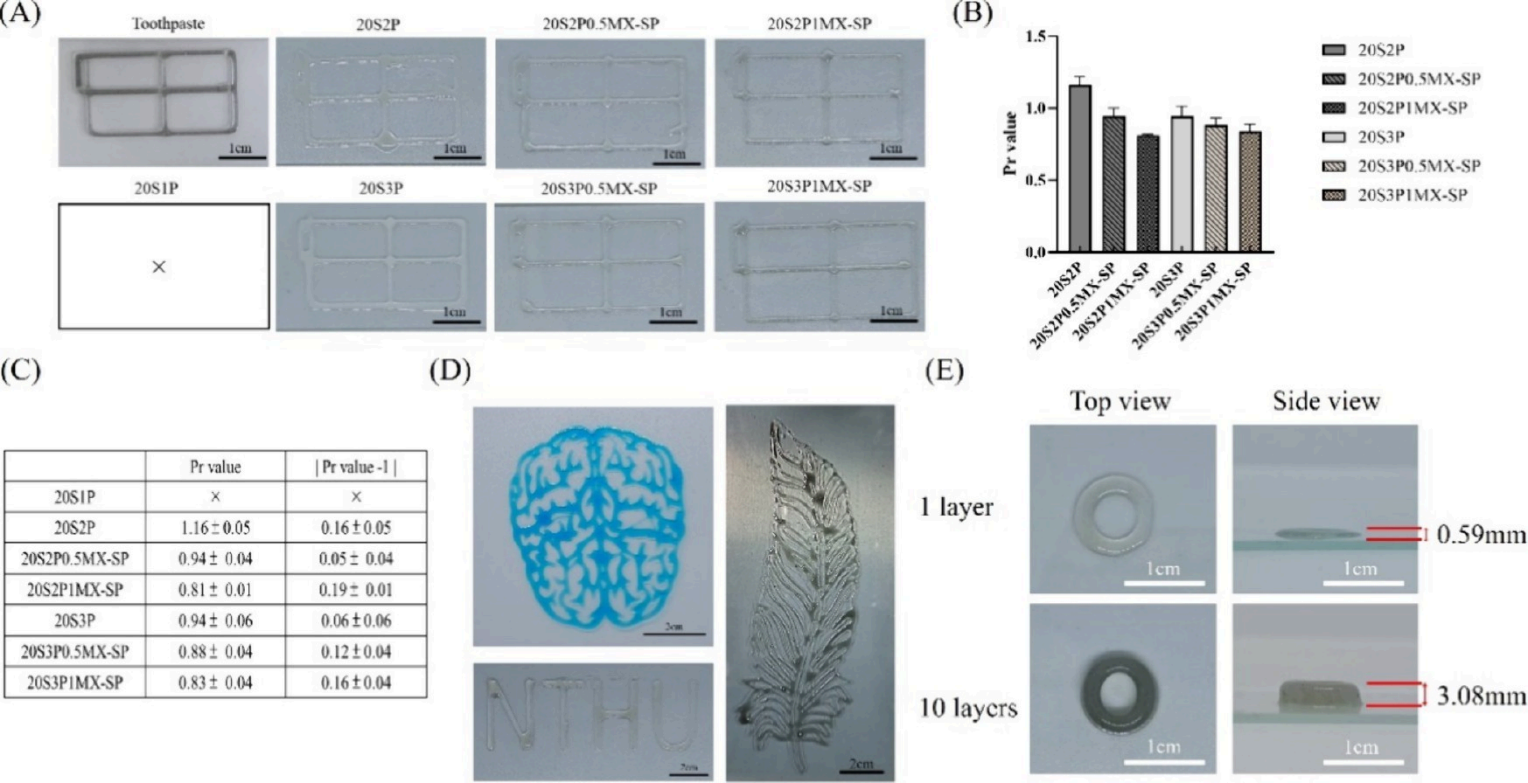
Bioink printability assessment and 3D printed constructs of series of SilMA/Pectin/MXene-SP bioinks (A) Bioink printability test. (B) Quantification of Pr value. (C) Pr value. (D) Complexity printability test. (E) Thickness of 20S3P1MX-SP bioink stacked in three and ten layers.

To compare the printability of the series of materials, we utilized a pneumatic dispensing system with toothpaste as a control group to print various SilMA/Pectin/MXene-SP bioinks as shown in Figure 4(A)-(C). The printability of the bioinks was assessed by calculating their Pr value, where a Pr value closer to 1 indicates better printability. A Pr value greater or less than 1 suggests that the bioink either collapses during extrusion or fails to extrude completely. The results indicate that the 20S1P bioink exhibited insufficient viscosity, preventing it from maintaining its shape after printing. In contrast, the groups with higher Pectin concentrations displayed better Pr values. Additionally, an increasing concentration of MXene-SP was associated with a slight decrease in the Pr value. Overall, these findings demonstrate that increasing the Pectin concentration enhances the printability of the bioink. Although MXene-SP may slightly affect extrusion, it still maintains the integrity of the printed structure within this concentration range. As shown in Figure 4(D), the 20S2P1MX-SP bioink can print intricate patterns, demonstrating its excellent printability and controllability. Furthermore, Figure 4(E) illustrates that the addition of 2% Pectin enhances the bioink’s stacking ability, allowing it to achieve a thickness of 3.08 mm after printing 10 layers.

### 3.8 Conductivity Analysis of the Series of SilMA-Based Bioinks

To investigate the impact of MXene-SP on the conductivity of SilMA-based hydrogels, we used electrochemical impedance spectroscopy (EIS) in a dual-electrode setup. This method allows for the evaluation of conductivity, charge transfer resistance, and diffusion properties by applying an alternating current across a range of frequencies and measuring the impedance response. Initially, to confirm whether the modified MXene-SP affects its conductivity, Figure S5 compares the 20S2P0.5MX and 20S2P1MX groups with those containing the modified MXene-SP. Figure S4(A) and Figure 4(A) shows the resulting Nyquist plot, with real impedance (Z’) on the *X*-axis (high to low frequency from left to right) and imaginary impedance (Z”) on the *Y*-axis. In this plot, the high-frequency semicircles represent electron and ion transport within the hydrogel, while the low-frequency linear regions reflect diffusion-limited behavior. As shown in Figure S4(A), the hydrogels containing MXene-SP and MXene exhibit smaller semicircles at high frequencies, indicating lower charge transfer resistance. Among hydrogels with the same composition, the MXene-SP group shows an even smaller semicircle radius compared to the MXene group. Additionally, Figure S4(B) demonstrates that at low frequencies, the impedance of the MXene group hydrogels is higher than that of the MXene-SP group hydrogels. Conductivity calculations presented in Figure S4(D) further reveal that the MXene-SP group exhibits better conductivity than the MXene group. In summary, the modified MXene-SP not only retains its conductivity but also demonstrates enhanced conductivity, likely due to its improved dispersibility.

**Figure 5:**
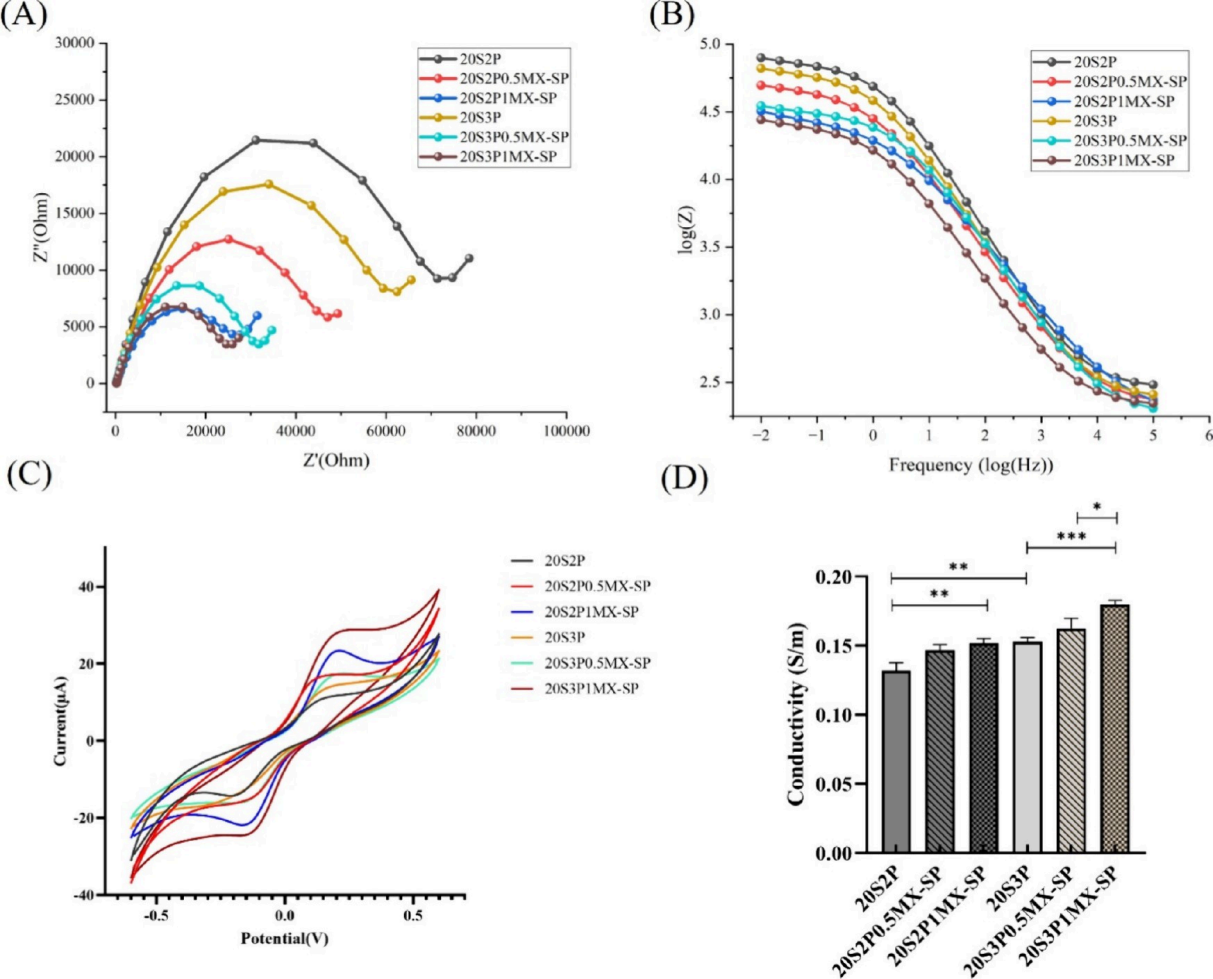
Electrochemical characteristics of series of SilMA/Pectin/MXene-SP hydrogels. (A) Nyquist plot. (B) Bode plot. (C) Cyclic voltammetry with scan range from −0.6 to 0.6 V at a scan rate of 50 mV/s. (D) Conductivity.

As shown in Figure. 5(A), comparing the 20S2P and 20S3P hydrogels, the smaller high-frequency semicircle for the 20S3P hydrogel suggests that increased pectin content enhances ionic properties, thereby reducing resistance. Furthermore, hydrogels containing MXene-SP exhibit even smaller semicircles, indicating a reduction in charge transfer resistance and suggesting improved electronic transport throughout the hydrogel matrix. Among all groups, the 20S3P1MX-SP hydrogel displays the smallest semicircle radius, indicating optimal electron transfer rates and superior conductivity. These findings suggest that incorporating MXene-SP, along with higher levels of Pectin, significantly improves the conductivity of SilMA-based hydrogels, highlighting their potential as electroactive biomaterials.

Figure 5(B) presents the Bode plot of the hydrogel series, illustrating impedance variations across a frequency range of 0.01 Hz to 100,000 Hz under an applied alternating voltage of 0.01 V. At lower frequencies, the hydrogels exhibit pronounced capacitive impedance characteristics. The results indicate that at 0.01 Hz, both 20S2P and 20S3P hydrogels display higher impedance compared to their MXene-SP-containing counterparts. Notably, the 20S3P1MX-SP hydrogel demonstrates the lowest impedance among all groups, aligning with observations from the Nyquist plot.

Figure 5(C) presents the cyclic voltammetry (CV) analysis results for the series of SilMA-based hydrogels, evaluated using a dual-electrode system. Potential scanning was performed from −0.6 to 0.6 V at a scan rate of 50 mV/s to compare the redox potentials of 20S2P and 20S3P hydrogels, both with and without MXene-SP. The data show that the redox potential increases with higher Pectin concentrations. Notably, the 20S3P1MX-SP hydrogel exhibits a higher redox current, reflecting improved electron transfer kinetics and an elevated concentration of electroactive species. These findings align with the electrochemical impedance data, further confirming that MXene-SP incorporation effectively reduces electron flow resistance within the hydrogel matrix.

Next, we measured the conductivity of the hydrogels using a dual-probe system under an alternating voltage of 0.01 V. Conductivity was calculated using a previously described formula, with results shown in Figure 5(D). The findings also indicate that adding both Pectin and MXene-SP enhances conductivity. Overall, increasing Pectin concentration reduces the charge transfer resistance of the hydrogels, likely due to Pectin’s negatively charged nature, which promotes electron mobility. Furthermore, hydrogels incorporating MXene-SP exhibit lower impedance compared to those containing unmodified MXene, suggesting that the enhanced dispersion of MXene-SP within the hydrogel matrix improves charge transfer and overall conductivity.

### 3.9 Biocompatibility Assessment of SilMA-Based Hydrogels via MTT Assay, Live/Dead Staining, and CCK-8 Assay

**Figure 6:**
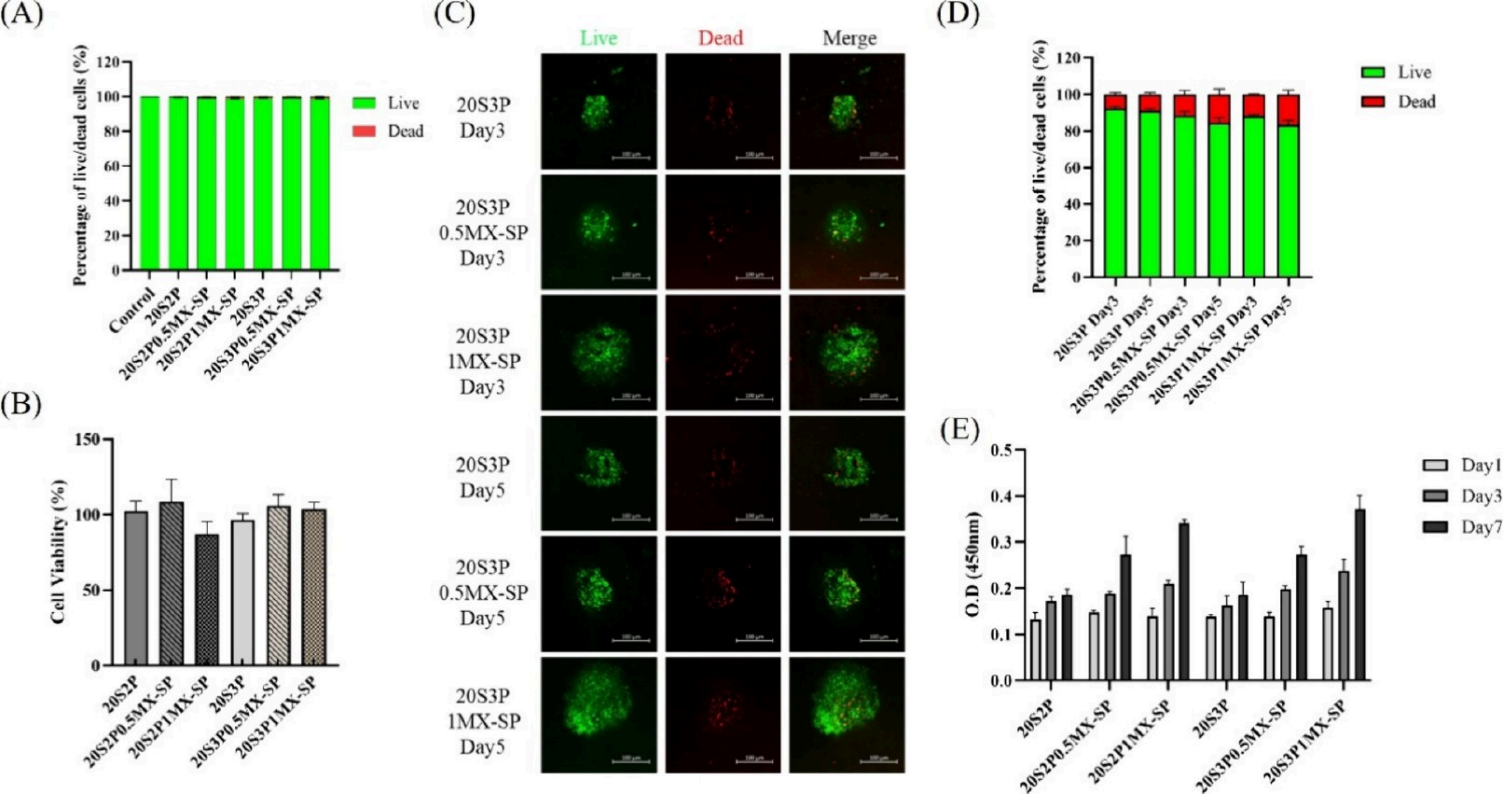
Biocompatibility test of L929 cells and NSCs spheroids encapsulated in series of SilMA/Pectin/MXene-SP hydrogel. (A) Live/Dead assay of L929 cells in series of hydrogels. (B) Cell viability assay of L929 cells in series of hydrogels. (C) Live and dead assay of NSCs spheroids in series of hydrogels (Scale bar = 100 μm). (D) Live/Dead fluorescence quantification and (E) CCK8 assay of NSC spheroids in hydrogels.

We further evaluated the biocompatibility of the hydrogels using the MTT assay, Live/Dead staining, and CCK-8 assay. Initially, we compared the biocompatibility of L929 cells within the series of hydrogels. The results presented in Figures 6(A) and 6(B) indicate that all SilMA/Pectin/MXene-SP hydrogel groups show no statistically significant differences compared to the control group, suggesting that the addition of MXene-SP does not adversely affect the inherent biocompatibility of the hydrogels.

Subsequently, NSCs were encapsulated in the hydrogels and cultured for three and 5 days. Live/Dead fluorescent staining was employed to observe the cell viability of NSCs. The quantitative results shown in Figure 6(D) reveal that while the addition of MXene-SP slightly affects the relative ratio of live to dead cells, all groups exhibited cell viability above 80% by day 5. Additionally, Figure 6(E) presents the CCK-8 analysis of NSC proliferation in the various hydrogel formulations over different culture durations. It is evident that with increasing concentrations of MXene-SP, significant cell proliferation occurs on both days 3 and 7. This enhancement in NSC proliferation may be attributed to the increased conductivity and the higher specific surface area provided by the two-dimensional MXene-SP, which may further promote NSC growth.

### 3.10 Differentiation of NSC Encapsulation in SilMA/Pectin/MXene Hydrogels

**Figure 7:**
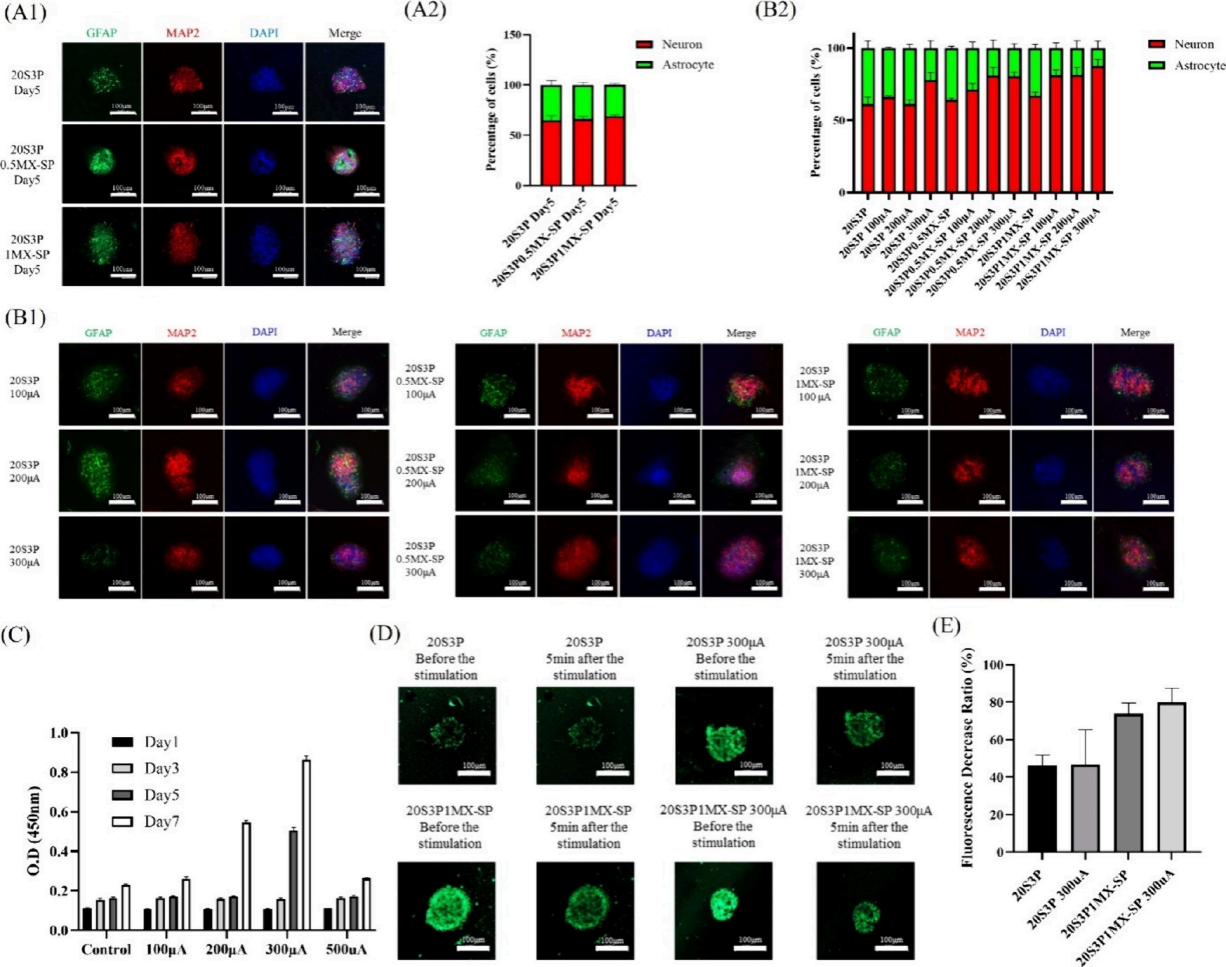
Differentiation and synaptic functionality of NSPCs encapsulated in series of hydrogels with/without electrical stimulation. (A1) and (B1) Immunofluorescence staining of MAP2 (red) and GFAP (green) expression of NSPC spheres in hydrogels with/without 5 days of electrical stimulation with different currents, respectively. (A2) and (B2) Quantification of the fluorescence intensity. (C) Proliferation of NSC spheroids on the 20S3P1MX-SP hydrogel after electrical stimulation at various currents. The assessment was conducted on days 1, 3, 5, and 7. (D) FM1–43 fluorescence of NSC spheres before and after high-potassium HBSS stimulation (Scale bar = 100 μm). (E) Quantification of FM1–43 fluorescence in NSC spheres before and after high-potassium HBSS stimulation.

As shown in Figure S5(B) and (C), the NSC spheroids, prior to being mixed with the bioink or embedded in the hydrogel, prominently express the neural stem cell marker Nestin and completely lack expression of the neuronal differentiation marker MAP2. There is also only minimal expression of the glial differentiation marker GFAP, indicating that the majority of the NSC spheroids before contact with the materials are primarily composed of NSCs. Figure 7(A) shows the immunostaining results for MAP2 and GFAP, along with the relative ratio quantification of MAP2/GFAP, after 5 days of NSC spheroid culture in three hydrogel types: 20S3P, 20S3P0.5MX-SP, and 20S3P1MX-SP. These results indicate that the NSCs efficiently differentiated into both neurons and astrocytes, forming complex 3D neural networks, which confirms that the hydrogel environment supports the differentiation of NSC spheroids. Quantitative analysis in Figure 7(A2) reveals a slight increase in the percentage of neurons in the 20S3P1MX-SP group compared to the group without MXene-SP. However, this difference is not statistically significant, suggesting that MXene-SP may not have a major impact on neuronal differentiation within the time frame studied.

### 3.11 Electrical Stimulation of NSC Encapsulation in SilMA/Pectin/MXene Hydrogels

According to previous studies, excessive astrocyte proliferation can lead to glial scar formation, which impedes nerve regeneration and intercellular signaling. Moderate electrical stimulation has been shown to promote the proliferation and differentiation of NSCs, contributing to reduced glial scar formation and enhanced nerve regeneration. To assess this, Figure 7(C) illustrates the analysis of NSC proliferation in the 20S3P1MX-SP hydrogel following electrical stimulation at different time points. The results show no significant increase in optical density (O.D.) values on the first and third days after electrical stimulation. However, by the fifth and seventh days, a marked increase in O.D. values was observed when the current was raised to 200 μA and 300 μA, indicating an enhancement in proliferation rates. The highest O.D. value was recorded at 300 μA; however, a decrease in O.D. was observed at 500 μA, suggesting that excessively high current levels may cause cellular damage. In summary, without electrical stimulation, the NSCs in the hydrogel exhibited slower growth, whereas electrical stimulation at 200 μA and 300 μA significantly promoted rapid proliferation and increased metabolic activity of the stem cells. Based on these findings, we chose to apply electrical stimulation at 100 μA, 200 μA, and 300 μA in subsequent experiments to examine their effects on neuronal differentiation.

To further investigate the effect of electrical stimulation on NSCs differentiation, we first varied the stimulation voltage. Figure S6(A) presents immunostaining analysis of NSCs cultured in the 20S3P, 20S3P0.5MX-SP, and 20S3P1MX-SP bioinks under continuous stimulation at fixed voltages of 80 mV, 100 mV, and 200 mV for 10 min daily over 5 days. Figure S6(B) quantifies the neuron/astrocyte differentiation ratio. The results indicate that NSC spheroids differentiated into neurons and astrocytes; however, no significant increase in the neuronal differentiation ratio was observed with higher voltages. Additionally, the combination of MXene-SP and fixed-voltage stimulation did not enhance neuronal differentiation. This could be attributed to the resistance within the hydrogel, which may reduce the current intensity when low voltages are applied. To address this, we adjusted our strategy to apply fixed-current stimulation to NSCs within the hydrogel, allowing precise control of the electrical stimulation experienced by the cells. Figure 7(B1) presents the immunofluorescence staining results, while Figure 7(B2) shows the quantification of cell differentiation in NSCs after five consecutive days of electrical stimulation at different currents (10 min each day). The results indicate that as the current increases, the relative proportion of MAP2-positive neurons also increases, reflecting a higher degree of neuronal differentiation. Additionally, the proportion of astrocytes significantly decreases with increasing concentrations of MXene-SP. In the group where NSC spheroids were embedded in 20S3P0.5MX-SP, neuronal differentiation percentages were 81.08 ± 4.63% at 200 μA and 80.49 ± 2.26% at 300 μA. In the 20S3P1MX-SP group, the neuronal differentiation percentage increased from 66.92 ± 4.62% without electrical stimulation to 81.19 ± 2.94% at 100 μA, and further increased to 87.57 ± 3.83% at 300 μA. These findings suggest that the addition of MXene-SP enhances the conductive properties of the hydrogel, promoting neuronal differentiation and connectivity.

Furthermore, to assess synaptic functionality, FM1–43, a lipophilic, water-soluble styryl dye, was used to observe depolarization responses, endocytosis, and vesicle release. Cells were stimulated with a high-potassium HBSS solution, and synaptic activity was monitored before and after stimulation. Figure 7(D) illustrates the synaptic activity of NSCs after 5 days of culture, comparing synaptic functionality with and without electrical stimulation. All groups exhibited a fluorescence decrease following high-potassium HBSS stimulation. The quantified fluorescence declines are shown in Figure 7(E). The fluorescence decrease in NSCs embedded in the 20S3P hydrogel was 46.09 ± 4.65%, while in the 20S3P1MX-SP group, the decrease was 73.74 ± 4.76%. In the electrically stimulated group, the fluorescence decrease was 79.99 ± 6.15%. In summary, combining the 20S3P1MX-SP hydrogel with moderate electrical stimulation significantly enhanced NSC proliferation, promoted neuronal differentiation, and improved synaptic functionality. These findings suggest that the conductive MXene-SP material, when coupled with electrical stimulation, facilitates enhanced synaptic transmission and endocytic activity, ultimately boosting neuronal activity. This highlights the potential of this conductive bioink for applications in neural tissue engineering.

To further investigate the behavior of NSCs after dissociating spheroids into single cells, the dispersed cells were encapsulated in the bioink and subjected to electrical stimulation to observe their proliferation and differentiation. The results were then compared with those of NSC spheroids. As shown in Figure S7(B), the proliferation trend of the dispersed cells under different current levels was similar to that of the spheroid cells. The highest O.D. value was observed at 300 μA, which increased with the duration of electrical stimulation. However, when the current reached 500 μA, a decrease in O.D. value was noted. Figure S7(A) and (C) examine the differentiation of dispersed cells in the hydrogel before and after electrical stimulation. The dispersed cells primarily differentiated into neurons within the hydrogel. From the quantification results in Figure S7(C), it is evident that in the 20S3P1MX-SP hydrogel containing MXene-SP, the neuron differentiation ratio was higher under 200 μA and 300 μA electrical stimulation compared to other groups. Remarkably, the differentiation ratio in these conditions even surpassed that of the spheroid cells. This outcome suggests that dispersed cells may achieve more uniform contact with the material and better sensing of the electrical stimulation, thereby promoting differentiation.

## 4 Conclusions

This study successfully developed a photo-cross-linkable SilMA/Pectin/MXene-SP bioink by modifying silk fibroin and MXene with GMA and soybean lecithin, respectively, and incorporating Pectin. The inclusion of MXene-SP enhanced the dispersion of MXene within the bioink, which resulted in improved shear-thinning properties as the Pectin concentration increased, thereby enhancing the material’s printability. The bioinks exhibited a porous structure that supports cell growth and nutrient exchange. Electrochemical testing demonstrated that MXene-SP possesses superior conductivity and biocompatibility compared to MXene. When NSCs were embedded in the SilMA/Pectin/MXene-SP bioink, a high proportion of neuronal differentiation was observed. Furthermore, electrical stimulation with a current of 300 μA for 10 min daily resulted in the highest proliferation rate in the bioink containing 1 mg/mL MXene-SP, with neuronal differentiation surpassing other groups and exhibiting enhanced synaptic functionality. In future work, we aim to further optimize the porosity, stacking ability, and extensibility of the bioink to improve its applications in tissue engineering, the construction of in vitro models, and the development and evaluation of new therapies.

## Data Availability

All data generated or analyzed during this study are included in this published article and its Supporting Information files.

## Supporting Information Available

Figure S1: Gelation and modulus of series of SilMA/Pectin with different concentrations of MXene-SP hydrogel under different exposure time; Figure S2: Gelation of series of SilMA/Pectin/MXene-SP hydrogels exposure on UV; Figure S3: Characterization of series of SilMA/Pectin/MXene-SP hydrogels; Figure S4: Electrochemical characteristics of MXene and MXene-SP at the same concentration of SilMA/Pectin; Figure S5: Images of NSC spheres and immunostaining prior to encapsulation with bioinks; Figure S6: Immunofluorescence staining of NSC spheres in series of hydrogels with electrical stimulation; Figure S7: Immunofluorescence staining of dissociated NSCs with electrical stimulation (PDF)
