# Supplementary material for: Innovative MXene/SilMA-Based Conductive Bioink for Three Dimensional Bioprinting of Neural Stem Cell Spheroids in Neural Tissue Engineering
Source: ACS Appl Mater Interfaces. 2025 Feb 7;17(7):10402–16. doi: 10.1021/acsami.4c19373 (PMC11843594; doi:10.1021/acsami.4c19373)
Supplement: Supplementary file 1 — am4c19373_si_001.pdf [file am4c19373_si_001.pdf]

Innovative MXene/SilMA-Based Conductive Bioink for 3D Bioprinting of NSC Spheroids in Neural Tissue Engineering

Yu-Chun Yeh[1], Pin-Yuan Chen[2], Ko-Ting Chen [3, #], and I-Chi Lee [1, *]

[1] Department of Biomedical Engineering and Environmental Sciences, National Tsing Hua University, Hsinchu, Taiwan

[2] Department of Neurosurgery, Chang Gung Memorial Hospital, Keelung branch, 20401, Taiwan

[3] Department of Neurosurgery, Chang Gung Memorial Hospital at Linkou, Taoyuan, Taiwan

* Corresponding author: I-Chi Lee

E-mail: iclee@mx.nthu.edu.tw

Tel: +886-3-5715131 ext 35525

[#] Co-Corresponding author: Ko-Ting Chen

Email: Chenkoting@gmail.com

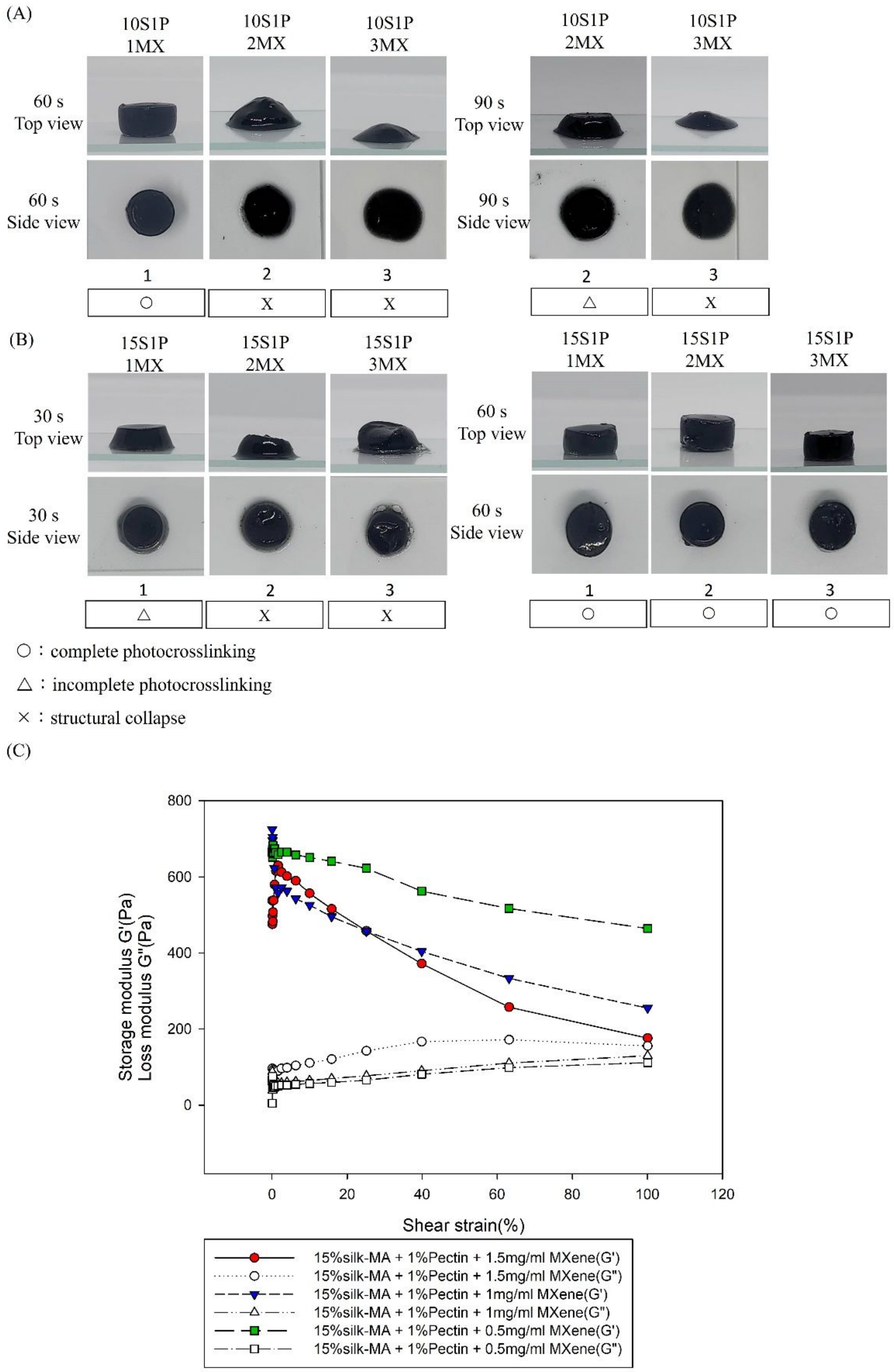

Figure S1. (A) Gelation of series of 10% SilMA/1% Pectin with different concentrations of MXene-SP hydrogels exposure on UV 60s and 90s. (B) Gelation of series of 15% SilMA/1% Pectin with different concentrations of MXene-SP hydrogels

exposure on UV 30s and 60s. (C) Storage modulus and loss modulus of 15%SilMA/1% Pectin with different concentrations of MXene-SP hydrogels at different frequencies.

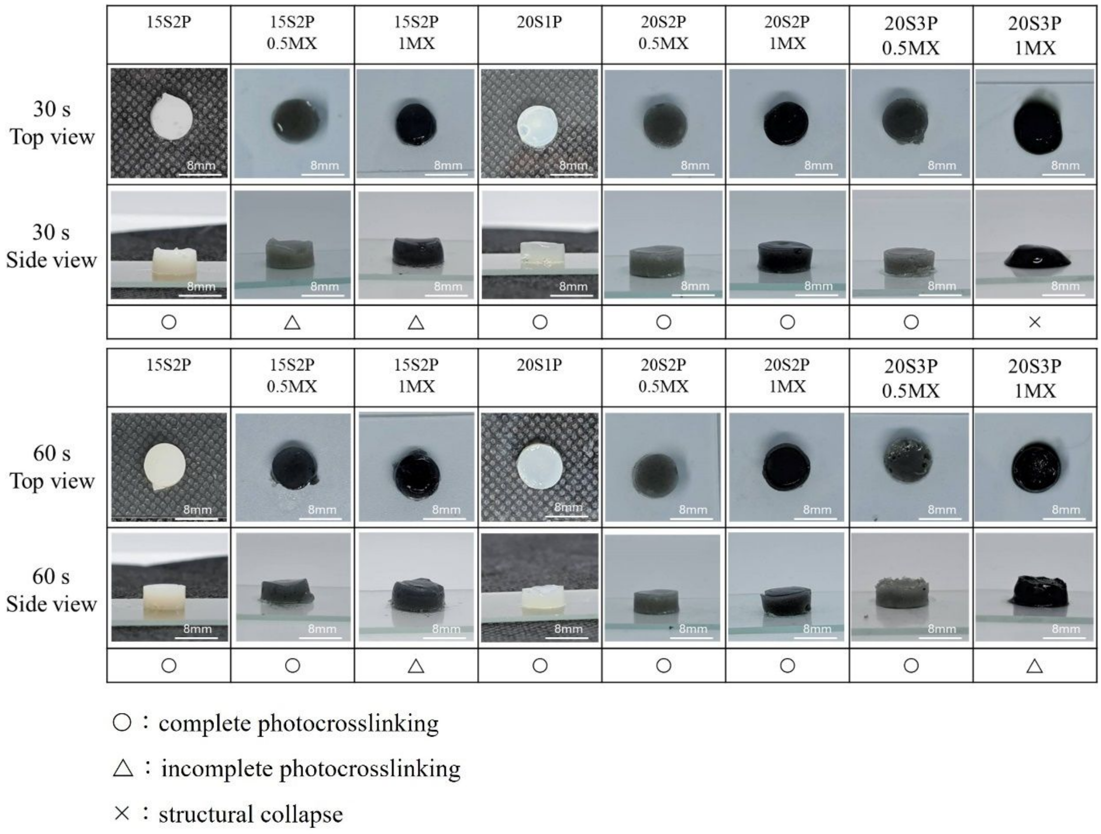

Figure S2. Gelation of series of SilMA/Pectin/MXene-SP hydrogels exposure on UV 30s and 60s.

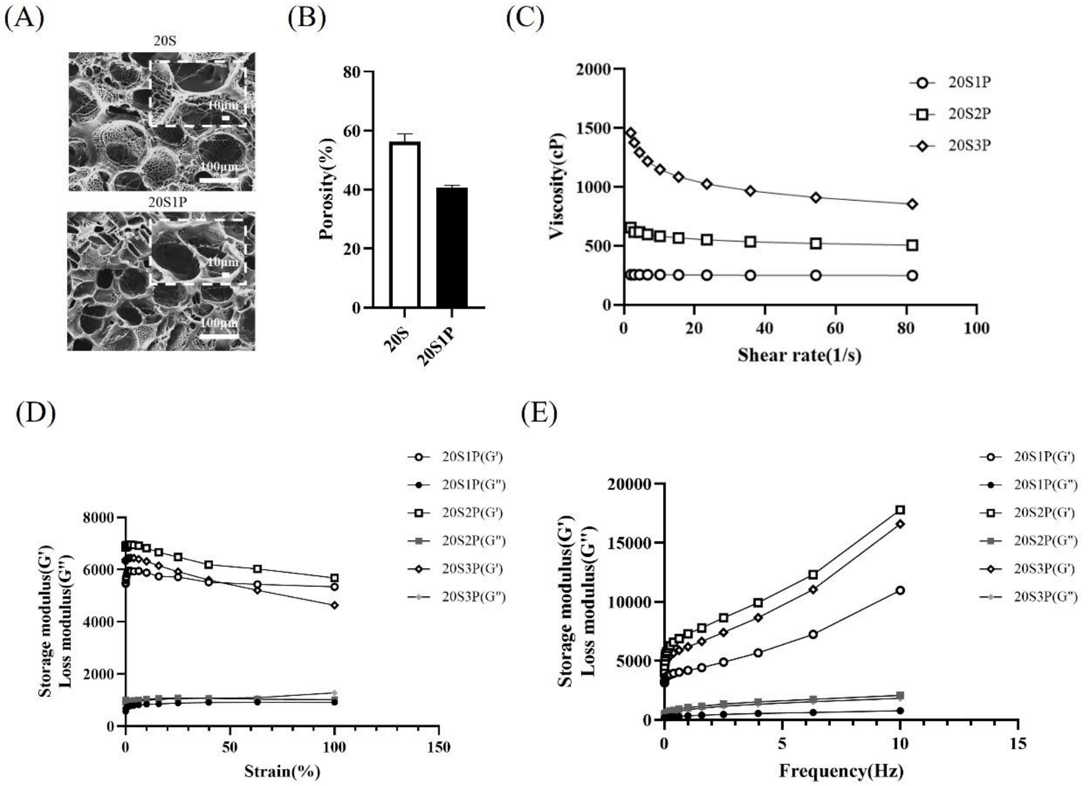

Figure S3. Characterization of series of SilMA/Pectin/MXene-SP hydrogels (A) SEM images of different composition of hydrogels. (B) Porosity of different composition of hydorgels. (C) Shear thinning different composition of SilMA/Pectin bioink. (D) Storage modulus and loss modulus of SilMA/Pectin hydrogels at different frequencies. (E) Strain sweeps of SilMA/Pectin hydrogels bioink at frequency of 10Hz.

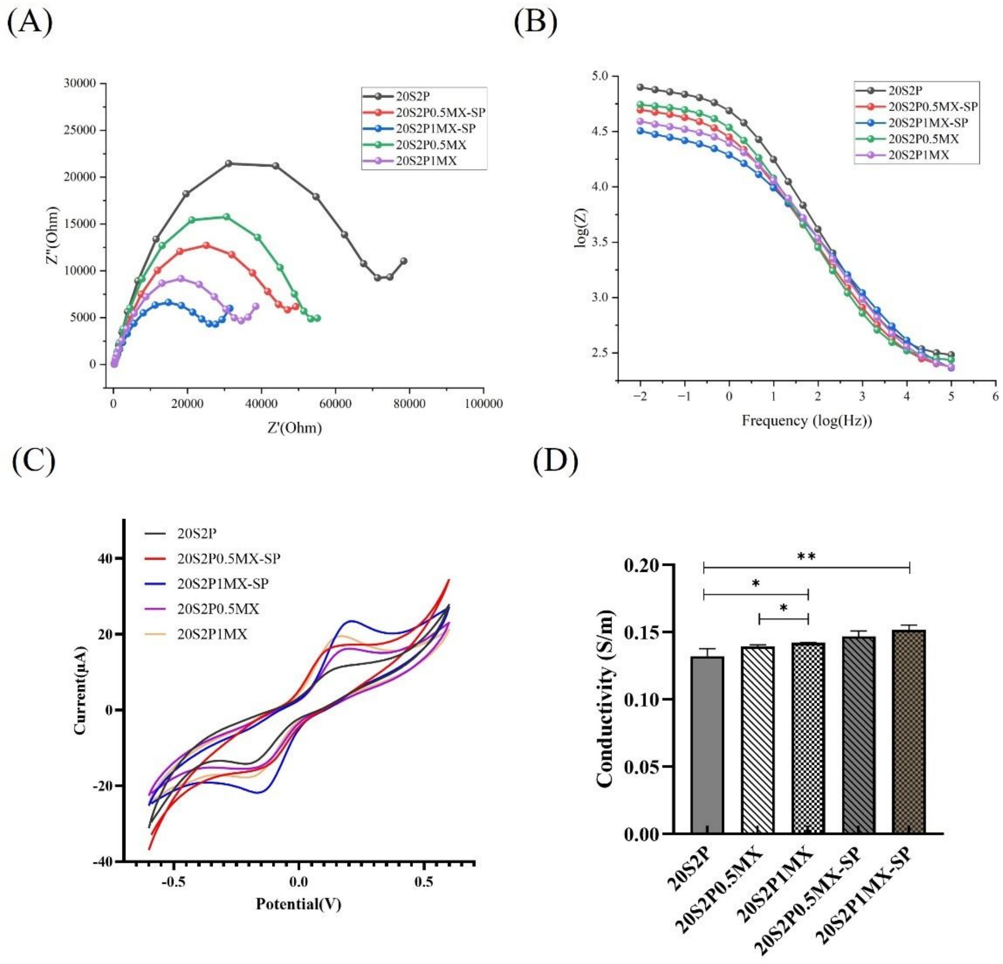

Figure S4. Electrochemical characteristics of MXene and MXene-SP at the same concentration of SilMA/Pectin. (A)Nyquist plot. (B) Bode plot. (C) Cyclic voltammetry with scan range from −0.6 to 0.6 V at a scan rate of 50 mV/s. (D) Conductivity.

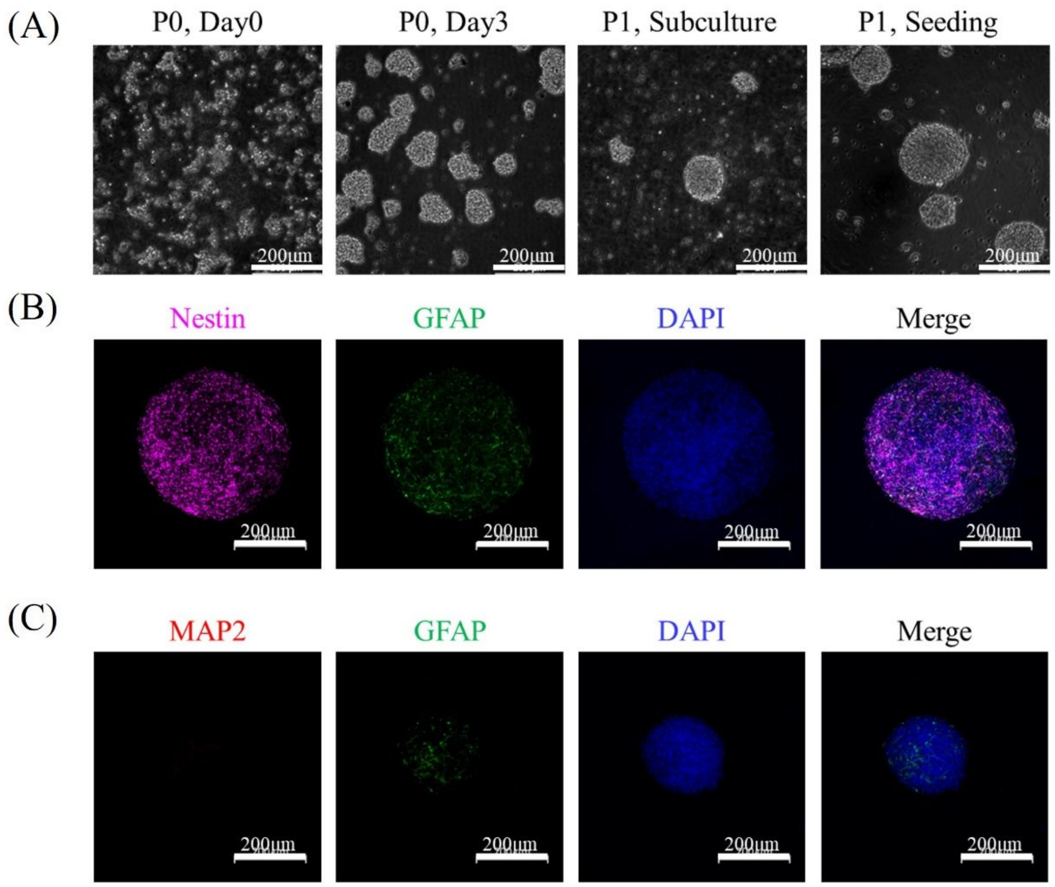

Figure S5. Images of NSC spheres and immunostaining prior to encapsulation with bioinks. (A) NSCs image before seeding. (B) Nestin and GFAP expression of NSCs. (C) MAP2 and GFAP expression of NSCs.

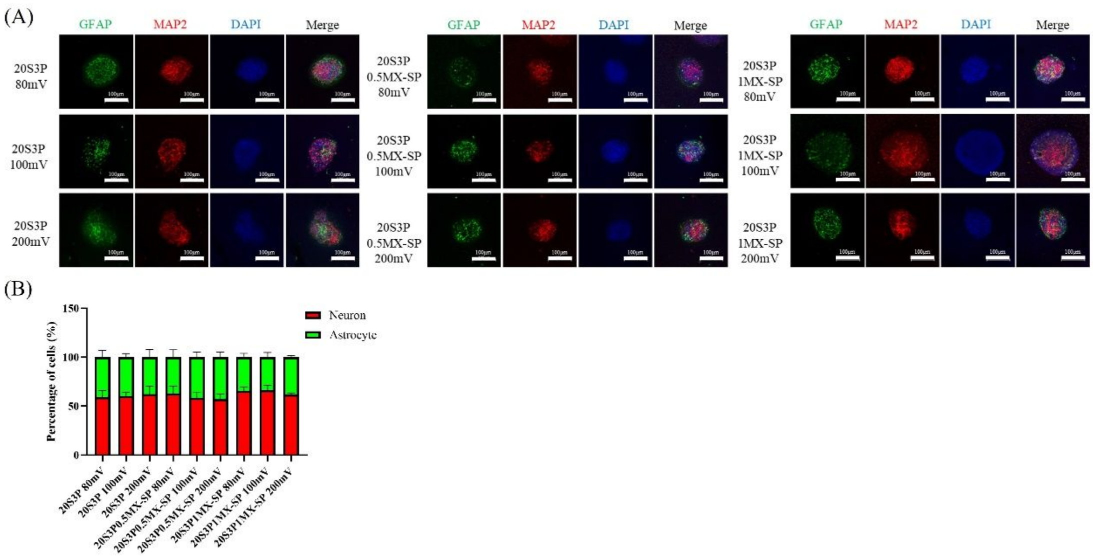

Figure S6. (A) Immunofluorescence staining of NSC spheres in series of hydrogels after 5 days of electrical stimulation with different voltages. (B) Quantification of the fluorescence intensity.

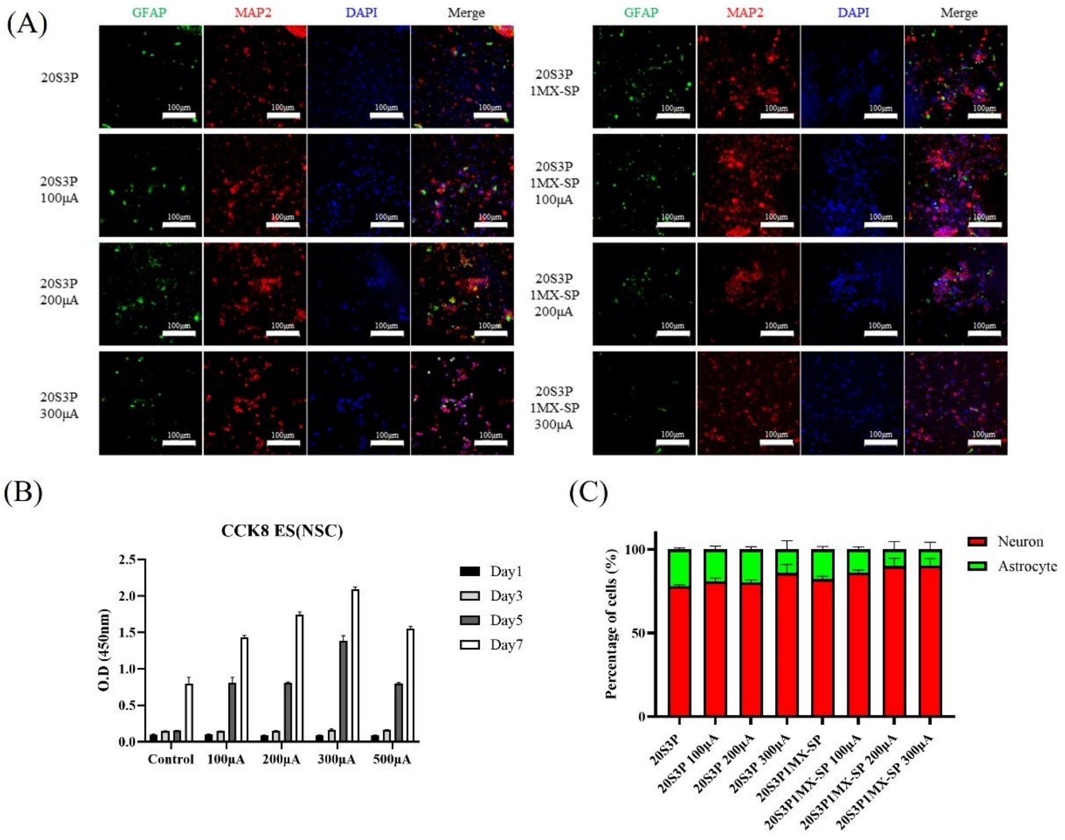

Figure S7. (A) Immunofluorescence staining of dissociated NSCs in hydrogels after 5 days of electrical stimulation with varying currents. (B) Proliferation of dissociated NSCs on the 20S3P1MX-SP hydrogel assessed on the first, third, fifth, and seventh days following electrical stimulation at different currents. (C) Quantification of fluorescence intensity.
